# A promoter toolbox for tissue-specific expression supporting translational research in cassava (*Manihot esculenta*)

**DOI:** 10.3389/fpls.2022.1042379

**Published:** 2022-12-20

**Authors:** Wolfgang Zierer, Ravi Bodampalli Anjanappa, Christian Erwin Lamm, Shu-Heng Chang, Wilhelm Gruissem, Uwe Sonnewald

**Affiliations:** ^1^ Biochemistry, Department of Biology, Friedrich-Alexander University Erlangen-Nuremberg, Erlangen, Germany; ^2^ Plant Biotechnology, Department of Biology, Eidgenössische Technische Hochschule (ETH) Zurich, Zurich, Switzerland; ^3^ Advanced Plant Biotechnology Center, National Chung Hsing University, Taichung, Taiwan

**Keywords:** cassava, biotechnology, promoter, storage root, parenchyma, phloem, xylem, tissue

## Abstract

There is an urgent need to stimulate agricultural output in many tropical and subtropical countries of the world to combat hunger and malnutrition. The starchy crop cassava (*Manihot esculenta*), growing even under sub-optimal conditions, is a key staple food in these regions, providing millions of people with food. Cassava biotechnology is an important technique benefiting agricultural progress, but successful implementation of many biotechnological concepts depends on the availability of the right spatiotemporal expression tools. Yet, well-characterized cassava promoters are scarce in the public domain. In this study, we investigate the promoter activity and tissue specificity of 24 different promoter elements in stably transformed cassava plants. We show that many of the investigated promoters, especially from other species, have surprisingly low activity and/or tissue specificity, but feature several promoter sequences that can drive tissue-specific expression in either autotrophic-, transport- or storage tissues. We especially highlight *pAtCAB1*, *pMePsbR*, and *pSlRBCS2* as strong and specific source promoters, *pAtSUC2*, *pMeSWEET1-like*, and *pMeSUS1* as valuable tools for phloem and phloem parenchyma expression, and *pStB33*, *pMeGPT*, *pStGBSS1*, as well as *pStPatatin Class I*, as strong and specific promoters for heterotrophic storage tissues. We hope that the provided information and sequences prove valuable to the cassava community by contributing to the successful implementation of biotechnological concepts aimed at the improvement of cassava nutritional value and productivity.

## Highlight

Providing expression tools for biotechnological applications by characterizing twenty-four promoter sequences in stably transformed cassava plants.

## Introduction

According to the latest Food and Agricultural Organization of the United Nations report (FAO et al., 2022), it is estimated that between 702 and 828 million people were affected by hunger in 2021 worldwide. The report states that most of the world’s undernourished people live in Asia (425 million people; approximately 9.1% of total population), while Africa is the region where the prevalence is the highest. 278 million people in Sub-Saharan Africa (SSA) suffer from chronic hunger. This is approximately 20% of the entire population (FAO et al., 2022). In addition, 399 million people are moderately food insecure, meaning that they don´t have regular access to sufficient food, even though they aren´t necessarily suffering from chronic hunger. The food insecurity situation has grown worse in the past years, mainly due to climate shocks, conflicts and economic slowdowns. The report concludes that it is equally important to diversify the economy and to simulate agricultural output (FAO et al., 2020).

The woody shrub cassava (*Manihot esculenta*) assumes a central role in (sub-)tropical countries, as one of the most important staple food crops. Especially in SSA, the crop is almost exclusively grown by smallholder farmers with limited resources for agricultural inputs, like industrial fertilizer. Even on poor soil, cassava can generate reasonable yields, is water efficient, and can withstand prolonged periods of drought. These factors, together with its flexible harvest time, make the crop very suitable for staple food production in a low input environment.

Half of the global annual cassava yield is produced in SSA, with Nigeria being by far the largest producer. Publically available FAO data (https://www.fao.org/faostat/en/#data/QCL; Inputs = Nigeria, Yield, Area harvested, 2000 – 2020) show that the cassava farming area in Nigeria has doubled between the years 2011 and 2012 and after, from 3 million to 6 million hectare. Around the same time, yield per area has dropped from approximately 10 metric tons per hectare to approximately 8 metric tons per hectare. These data show an overall increase in yield for Nigeria, which is mostly attributed to increased land use but not to increased productivity per area, which has in fact declined. Increasing the countries total cassava yield by further increasing land use does not seem to be a suitable solution for the individual smallholder farmers. Ideally, increases in yield would come from improvements to productivity per area, or in other words, more efficient farming methods and more high-yielding cassava varieties.

Alongside cassava breeding, biotechnology might be one of the tools that can contribute to increasing cassava yield. In the recent years, several biotechnological improvements have been realized in this important crop, especially concerning nutritional improvements and virus resistance. Some notable examples include the improvement of cassavas vitamin B6 content, iron and zinc content, and plant resistance to cassava mosaic virus and cassava brown streak virus (Li et al., 2015; Narayanan et al., 2019; Narayanan et al., 2021). A recent report from Chavarriaga-Aguirre et al. (2016) summarizes several of these improvements. However, the authors rightfully note that all transgenic cassava plants are stuck in the proof of concept stage and more translational research needs to happen to get these plants into the hands of farmers. Transgenic concepts need to move out of the laboratory into the field and be tested in multi-year and multi-location trials (Chavarriaga-Aguirre et al., 2016).

The “Cassava Source-Sink” project (https://cass-research.org/) focusses on cassava translational research and aims to improve cassava yield through breeding and biotechnology. Yield traits are typically polygenic traits, depending on the interaction of many genes. Flux through biochemical pathways is often coordinated with that of competing pathways, therefore, effective metabolic engineering will only be achieved by controlling multiple genes of the same, or interconnected, pathways (Halpin, 2005). Recent advances in cloning technologies (e.g. Golden Gate) and declining prices for DNA synthesis are supporting multigene approaches. Sonnewald et al. (2020) recently outlined a strategy towards cassava yield improvement by combining metabolic source-, transport-, and sink- improvements into transgenic cassava plants with subsequent field performance testing. However, it has to be noted that realizing such transgenic multigene approaches and their translation into the field comes with additional challenges like complicated international logistics, high regulatory effort, and long time-lines for cassava transformation and field-testing.

Another challenge for the translation of transgenic yield improvements to cassava is the availability of established expression tools, especially tissue-specific promoter elements. Since the successful implementation of a transgenic concept often needs very cell-/tissue-specific promoters or a combination of several promoters with a particular strength and specificity, the characterization of such promoters becomes essential. This is especially true for yield traits, where likely more than one gene needs to be transferred. To name just three examples from a large body of literature: (i) Root growth, drought resistance and overall yield could be improved by specifically expressing a cytokinine oxidase in the root elongation zone in thale cress, tobacco, barley, and chickpea. Due to the cell-/tissue-specific expression, the inhibitory effect of cytokinine on side-root formation was removed without negatively affecting the elongation root growth from the root apical meristem, leading to an overall larger root system (Werner et al., 2010; Ramireddy et al., 2018; Khandal et al., 2020). (ii) In field-grown maize, yield improvements could be demonstrated by expressing a trehalose-6-phosphatase specifically in maize ears, leading to an increased assimilate supply for this specific plant part (Nuccio et al., 2015). (iii) Recently, a couple of successful multigene stack approaches for yield improvement have been published for thale cress, tobacco, or potato, each requiring at least three well-performing promoters (Jonik et al., 2012; Kromdijk et al., 2016; South et al., 2019).

Cassava promoters, which have in fact been tested and confirmed in cassava itself, are quite scarce. Due to the difficult and lengthy cassava transformation process, cassava promoters have often been characterized by using heterologous expression systems in the past [e.g. Arango et al. (2010); Suhandono et al. (2014)], potentially resulting in incorrect promoter assessments. Unfortunately, there seems to be only a small amount of literature characterizing cassava promoters with stably transformed cassava plants (Zhang et al., 2003; Beltran et al., 2010; Koehorst-van Putten et al., 2012; Oyelakin et al., 2015; Wilson et al., 2017; Mehdi et al., 2019). Zhang et al. (2003); Beltran et al. (2010), and Oyelakin et al. (2015) have described promoter sequences from *Manes.12g132900* and *Manes.12g062400*, from a glutamic-acid-rich protein Pt2L4, or from the cassava vein mosaic virus, respectively. However, all four promoters displayed a rather ubiquitous expression pattern with slight preference for particular tissues. Several promoters have also been analyzed in the frame of a global cassava expression study (Wilson et al., 2017), although unfortunately not in great detail. Mehdi et al. (2019) has analyzed the specificity of the thale cress *SUC2/SUT1* promoter in cassava *via* stably transformed promoter-GFP plants, demonstrating its phloem companion cell specificity. While the leave vasculature was not visible in the *pSUC2::GFP* plants, presumably because of the detection limit, the *pSUC2::GUS* plants presented here, confirm its activity along the entire phloem.

Since storage roots are the prime product of cassava, storage root specific promoters are particularly useful for cassava trait improvement and multiple studies have highlighted the specificity of the potato *Patatin Class I* promoter [e.g. Ihemere et al. (2006); Zidenga et al. (2012); Vanderschuren et al. (2014); Gaitan-Solis et al. (2015); Li et al. (2015); Zhou et al. (2017); Beyene et al. (2018); Wang et al. (2018); Narayanan et al. (2019)]. In addition, Koehorst-van Putten et al. (2012) suggested *pMeGBSS1* as a storage-root specific promoter for cassava, based on the analysis of promoter-luciferase plants. Unfortunately, storage root specificity for the *MeGBSS1* promoter sequence could not be confirmed in this study. More well described promoters are needed to support cassava biotechnology approaches. In addition to promoter sequences specific for autotrophic tissues, promoters specific for heterotrophic tissues like phloem or storage parenchyma, or promoters with very cell-specific expression patterns, will be most valuable.

In this study, we share our findings about the promoter activity and specificity of 24 promoter elements in total. Initially, we characterized 10 promoters with a combination of expression data from field-grown, multigene construct lines, as well as dedicated promoter-gus plants and discovered a surprisingly low activity and/or specificity for the majority of these promoters. Consequently, we tested 14 additional promoter sequences *via* stably transformed promoter-gus plants with the goal to obtain a selection of tissue-specific promoters for autotrophic-, transport-, and heterotrophic storage tissues. We recommend a subset of tissue-specific promoters in the hope that these tools will also help other groups to improve their cassava research and translational work.

## Results

### Activity and tissue specificity of ten promoters in transgenic, field-grown cassava plants

**Figure 1:**
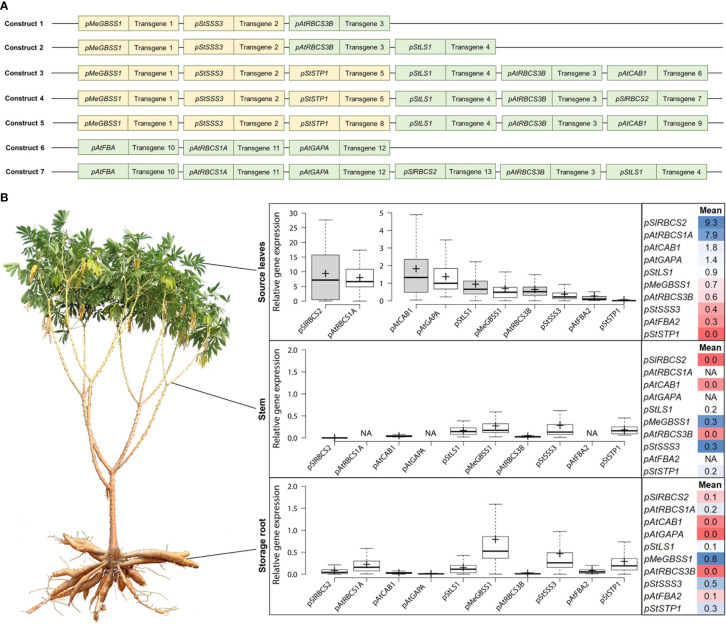
Summary of the approximate promoter activity of ten different promoters in source leaves, stem, and storage root. **(A)** Composition of the seven multigene constructs analyzed for gene expression of the individual target genes (target genes not shown). Orange indicates promoter choice for desired expression in heterotrophic tissues, green indicates promoter choice for desired expression in autotrophic tissues. **(B)** The relative gene expression (normalized to *MeGAPDH*) of different transcripts was determined and the data was used to infer the approximate activity of the promoter element controlling its expression. Field-grown cassava plants were used to sample fully exposed source leaves (in the afternoon), stem pieces at the lower end of the first branching point, and storage root material from the two thickest storage roots per plant.

In an attempt to improve cassava yield by altering different parts of cassava metabolism simultaneously, transgenic cassava plants expressing various combinations of metabolically active genes, altering photosynthetic-, transport-, and storage metabolism, were created and field-tested at NCHU experimental station in Taichung, Taiwan. The plants contained one of seven different multigene constructs, each construct combining three to six different target genes, with the respective target genes always being controlled by the same promoter (**Figure 1A**). The ten promoter elements used in these constructs were untested for their performance in cassava prior to their use and were initially selected due to their described activity in other plant species. The promoters were expected to mediate specific expression of target genes for autotrophic (also called “source” tissues, following the carbohydrate-based definition) or heterotrophic (also called “sink” tissues, following the carbohydrate-based definition) tissues (**Table 1**).

**Table 1:** Summary of 10 promoters assayed via their GUS staining pattern and/or transgene expression in field-grown, transgenic cassava plants.

Name	Code	Source organism	Gene/Closest NCBI identifier	Reference	Expected tissue	Analyzed by	Results in Fig.
*CHLOROPHYLL A/B-BINDING PROTEIN 1*	*AtCAB1*	*Arabidopsis thaliana*	*At1g29930*	Mitra et al., 2009; Engler et al., 2014	Autotrophic tissues	Histology/qPCR	1 & 3
*FRUCTOSE-BISPHOSPHATE ALDOLASE 2*	*AtFBA2*	*Arabidopsis thaliana*	*AT4G38970*	Lu et al., 2012	Autotrophic tissues	qPCR	1
*GLYCERALDEHYDE 3-PHOSPHATE DEHYDROGENASE SUBUNIT A*	*AtGAPA*	*Arabidopsis thaliana*	*AT3G26650*	Shih et al., 1992	Autotrophic tissues	qPCR	1
*LEAF-SPECIFIC 1*	*StLS1*	*Solanum tuberosum*	*X04753.1*	Stockhaus et al., 1987; Engler et al., 2014	Autotrophic tissues	Histology/qPCR	1 & 3
*RIBULOSE BISPHOSPHATE CARBOXYYLASE SMALL SUBUNIT 1A*	*AtRBCS1A*	*Arabidopsis thaliana*	*AT1G67090*	Dedonder et al., 1993	Autotrophic tissues	qPCR	1
*RIBULOSE BISPHOSPHATE CARBOXYYLASE SMALL SUBUNIT 2*	*SlRBCS2*	*Solanum lycopersicum*	*X66069.1*	Kyozuka et al., 1993; Engler et al., 2014	Autotrophic tissues	qPCR	1
*RIBULOSE BISPHOSPHATE CARBOXYYLASE SMALL SUBUNIT 3*	*AtRBCS3B*	*Arabidopsis thaliana*	*At5g38410*	Dedonder et al., 1993; Engler et al., 2014	Autotrophic tissues	Histology/qPCR	1 & 3
*STARCH PHOSPHORYLASE 1*	*StSTP1*	*Solanum tuberosum*	*X73684.1*	Sonnewald et al., 1995	Heterotrophic tissues	Histology/qPCR	1 & 3
*GRANULE-BOUND STARCH SYNTHASE 1*	*MeGBSS1*	*Manihot esculenta*	*Manes.02G001000*	Koehorst-van Putten et al., 2012	Heterotrophic tissues	Histology/qPCR	1 & 3
*SOLUBLE STARCH SYNTHASE 3*	*StSSS3*	*Solanum tuberosum*	*X95759.1*	Abel et al., 1996	Heterotrophic tissues	Histology/qPCR	1 & 3

Promoters expected to be specific for photosynthetic tissues are highlighted in light green and promoters expected to be specific for heterotrophic storage organs are highlighted in orange.

Over 400 field-grown plants, representing 7 different constructs and 84 transgenic events were analyzed for their transgene expression, to get an insight into the promoter performance controlling the respective expression. Cassava source leaves, stems, and storage root samples were analyzed *via* quantitative RT-PCR and the results were summarized for each promoter (**Figure 1B**).

A very strong source leaf expression, although with large variation, was observed for the transcripts controlled by the promoters of *pSlRBCS2* (739 bp) and *pAtRBCS1A* (1175 bp). Their transcripts were approximately 4-5 times more abundant than the transcript controlled by the next strongest leaf promoter *pAtCAB1* (779 bp). High abundance in source leaves was also observed for the transcripts controlled by *pAtGAPA* (1008 bp) and *pStLS1* (1497 bp). Moderate transcript abundance in source leaves was detected for *pMeGBSS1* (1163 bp) and *pAtRBCS3B* (800 bp). Low levels in source leaves were found for the transcripts controlled by *pStSSS3* (1015 bp) and *pAtFBA2* (1000 bp), while no transcripts were found for *pStSTP1* (2081 bp).

In the heterotrophic organs, moderate to low levels were determined for the transcripts controlled by *pMeGBSS1*, *pStSSS3*, and *pStSTP1*. Low levels were found for *pAtRBCS1A* and residual levels were found in heterotrophic tissues for the transcripts controlled by the leaf-promoters *pSlRBCS2*, *pStLS1*, and *pAtFBA2*.

Based on the transcript abundance observed in the different organs (**Figure 1**), the promoters of *pSlRBCS2* and *pAtRBCS1A* appear to be very active in source leaves, although *pAtRBCS1A* seems to have a low-level activity in sink organs, as well. The promoters of *AtCAB1* and *AtGAPA* were characterized by high and very specific source leaf expression, while the promoters of *pAtRBCS3B* and *pAtFBA2* appeared rather weak. The promoter of *StLS1* also showed weak activity in source leaves with additional residual activity in sink organs.

The promoters of *MeGBSS1*, *StSSS3*, and *StSTP1* were expected to be specific for heterotrophic organs. However, the abundance of transcripts controlled by the promoters of *MeGBSS1* and *StSSS3* was comparable between the three tissues tested. Only *pStSTP1* seems to have a specific activity for heterotrophic organs (**Figure 1**). According to the PCR results, all three of these promoter sequences resulted in rather weak activity.

### Histological characterization of the analyzed cassava tissues

**Figure 2:**
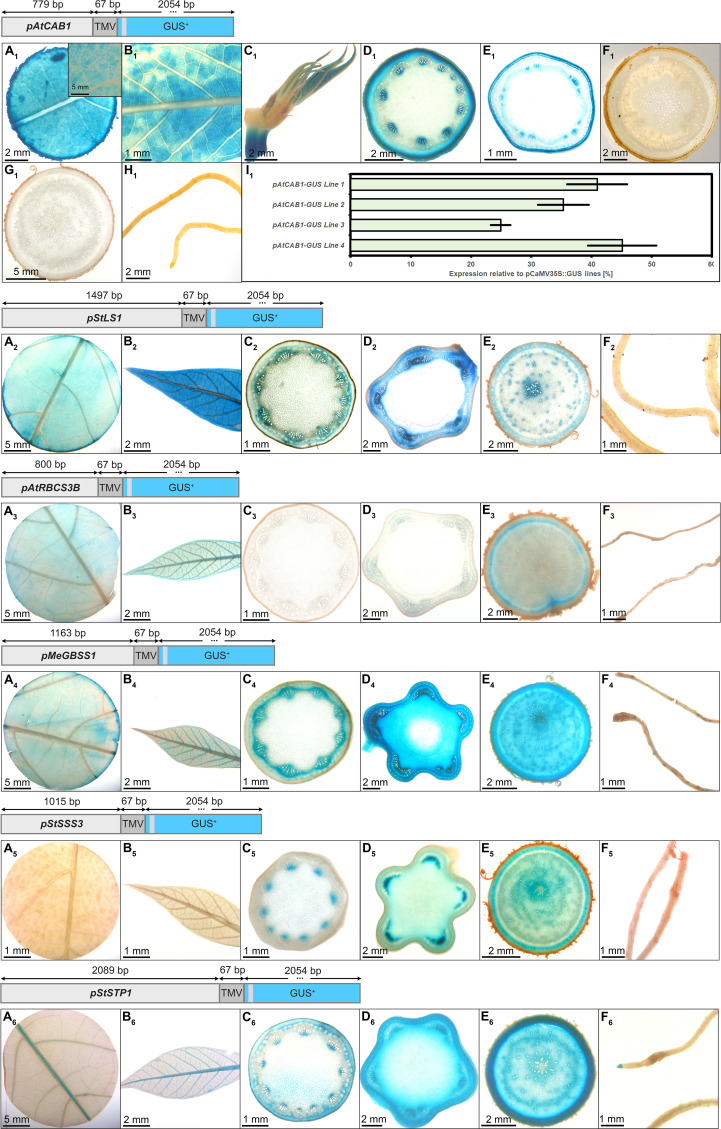
Representative GUS staining pattern of at least three events from *pAtCAB1*, *pStLS1*, *pAtRBCS3B*, *pMeGBSS1*, *pStSSS3*, and *pStSTP1* promoter-reporter plants. *pAtCAB1::GUS* = **A_1_**) Source leaf (Inlay = Close-up), **B_1_**) Sink leaf, **C_1_**) Emerging leaves, **D_1_**) Petiole crosssection, **E_1_**) Upper stem cross-section, **F_1_**) Lower stem cross-section, **G_1_**) Storage root cross-section, **H_1_**) Fibrous roots, **I_1_**) GUS expression levels of four *pAtCAB1::GUS* lines relative to three pCaMV35S::GUS lines in %. *pStLS1::GUS* = **A_2_**) Source leaf, **B_2_**) Sink leaf, **C_2_**) Petiole cross-section, **D_2_**) Upper stem cross-section, **E_2_**) Storage root cross-section, **F_2_**) Fibrous roots. *pAtRBCS3B::GUS* = **A_3_**) Source leaf, **B_3_**) Sink leaf, **C_3_**) Petiole cross-section, **D_3_**) Upper stem cross-section, **E_3_**) Storage root cross-section, **F_3_**) Fibrous roots. *pMeGBSS1::GUS* = **A_4_**) Source leaf, **B_4_**) Sink leaf, **C_4_**) Petiole cross-section, **D_4_**) Upper stem cross-section, **E_4_**) Storage root cross-section, **F_4_**) Fibrous roots. *pStSSS3::GUS* = **A_5_**) Source leaf, **B_5_**) Sink leaf, **C_5_**) Petiole cross-section, **D_5_**) Upper stem cross-section, **E_5_**) Storage root cross-section, **F_5_**) Fibrous roots. *pStSTP1::GUS* = **A_6_**) Source leaf, **B_6_**) Sink leaf, **C_6_**) Petiole cross-section, **D_6_**) Upper stem cross-section, **E_6_**) Storage root cross-section, **F_6_**) Fibrous roots. Plants were either grown on the field at NCHU experimental station Taichung, Taiwan or in a greenhouse in Erlangen, Germany. Tissues from approximately 3-month-old cassava plants were used.

For six (*pAtCAB1*, *pStLS1*, *pAtRBCS3*, *pMeGBSS1*, *pStSSS3*, *pStSTP1*) of the ten promoters included in the multigene construct plants and tested for their gene expression in the field (**Figure 1**), dedicated promoter-GUS plants were created, as well (**Figure 2**). For the analysis of promoter-GUS cassava plants, up to seven different tissues have been sampled and subjected to staining and microscopy: Emerging leaves, developing leaves, fully developed leaves, petioles, upper stem sections, lower stem sections, storage root sections, and fibrous roots (**Figure S1**). Emerging and developing leaves are characterized by brownish color and were termed “sink” leaves (defined as leaves that have a net import of carbon), while green, fully expanded leaves were considered “source” leaves (defined as leaves with net export of carbon).

The respective reporter plants displayed GUS staining in different tissues and cell types. To define these cell types, counterstaining with toluidine blue was performed and the results summarized in **Figure S1**. Source- and sink leaves can easily be divided into vascular bundles and mesophyll cells (**Figures S1A, B**). In petioles, the collenchyma, the sclerenchyma, the phloem, protoxylem/xylem parenchyma, pith parenchyma, and the pith cells can be differentiated from outside to inside (**Figure S1C**). Stem tissues are characterized by collenchyma, sclerenchyma, phloem, vascular cambium, and varying degrees of secondary xylem and pith tissue, depending on the position of the stem (**Figures S1D, E**). Especially the lower, heterotrophic stem tissues display increasing levels of secondary xylem tissues, consisting of xylem fibers, water-transporting xylem vessels, and starch-storing xylem parenchyma cells (**Figure S1E**). Storage roots have periderm tissue, the cork cambium, phelloderm/phloem parenchyma, phloem, vascular cambium, and xylem cells from outside to inside. Alongside xylem vessels, the xylem tissue is mostly dominated by starch-storing xylem parenchyma cells in storage roots (**Figure S1F**). Lower stems and storage roots, both heterotrophic starch-storing tissues, are overall similar and both tissues are characterized by many vascular rays, ensuring the connection of assimilate- and water transport systems, despite the increasing distance through the formation of secondary xylem during secondary growth (**Figures S1E, F**).

### Analysis of promoter-GUS plants matching the field-tested multigene construct plants

In the *pAtCAB1::GUS* events, staining was observed in the mesophyll of source leaves, sink leaves and newly emerging leaves (**Figures 2A1–C1**). Petiole and upper stem cross-sections displayed staining in collenchyma, outer parenchyma, and protoxylem areas (**Figures 2D1, E1**). Lower stem sections, storage roots and fibrous roots were completely devoid of GUS staining (**Figures 2F1–H1**). Therefore, the chosen promoter element of *AtCAB1* can drive expression in autotrophic cassava tissues without activity in heterotrophic plant parts. To determine the approximate expression strength of these promoter elements in the reporter plants, we determined the relative expression level of the different lines and compared them to the relative expression levels of *pCaMV35* as determined in three *pCaMV35S::GUS* lines. The promoter of *CaMV35S* is ubiquitously active in cassava as well (**Figure S2**) and its expression strength was used as a tangible reference point throughout the study. The promoter of *AtCAB1* showed approximately 25% to 45% activity compared to the promoter element of *CaMV35S*, respectively (**Figure 2I1**). Since *pCaMV35S* is a well-documented, strong promoter, the promoter of *AtCAB1* can drive specific and reasonably strong expression in the autotrophic tissues of cassava, which is in line with the field expression results displayed in **Figure 1**.

The promoter of *pStLS1* displayed the expected staining in the source- and sink leaves (**Figures 2A2, B2**). However, it also displayed staining in phloem and xylem tissues of petioles, stems and storage roots (**Figures 2C2, E2**). Only the fibrous roots were devoid of staining (**Figure 2F2**). This staining pattern matches the expression results (**Figure 1**), demonstrating that *pStLS1* has activity in both source- and sink tissues in cassava.

Similar to the low transcript levels observed for *pAtRBCS3B* (**Figure 1**), rather faint staining patterns were observed for *pAtRBCS3B::GUS*. Staining was seen in source- and sink leaves (**Figures 2A3, B3**), as well as, unexpectedly, in the storage root cambium region (**Figure 2E3**). It seems that *pAtRBCS3B* is not a good promoter for strong or specific expression in cassava.

The promoter of *pMeGBSS1* was expected to be sink specific (Koehorst-van Putten et al., 2012). However, activity in both source- and sink tissue was observed. Source leaves (**Figure 2A4**) and sink leaves (**Figure 2B4**) were stained, and strong staining was seen in the phloem area of the petiole (**Figure 2C4**). Besides the stem pith, all cell types of stems and storage roots were stained (**Figures 2D4, E4**). Fibrous roots were devoid of staining (**Figure 2F4**). Although the staining in stems and storage roots appears to be stronger compared to the other tissues, the inferred promoter activity from the expression results (**Figure 1**) suggest a rather equal activity between source- and sink. In any case, the promoter was not storage root specific in our experiments.

In contrast to the expression results (**Figure 1**), indicating comparable source- and sink activity, the *pStSSS3::GUS* plants displayed a staining specific for heterotrophic tissues. Staining was observed in the protoxylem of petioles (**Figure 2C5**) and stems (**Figure 2D5**), in the phloem and xylem areas of the storage root (**Figure 2E5**), but not in the fibrous roots (**Figure 2F5**). Since the promoters used in the multigene constructs (**Figure 1**) can potentially be influenced by neighboring promoters, *pStSSS3* might indeed be specific for heterotrophic organs. However, it does not seem to display a strong activity.

The promoter of *StSTP1* displayed a weak but sink-specific behavior in the multigene construct plants (**Figure 1**). A matching staining pattern was observed in the promoter-GUS plants. The activity in source- and sink leaves was confined to the vasculature (**Figures 2A6, B6**). Petioles showed staining in the protoxylem and outside the sclerenchyma (**Figure 2C6**). While fibrous roots displayed no staining besides the root tip (**Figure 2F6**), most staining was observed in the stems (**Figure 2D6**) and storage roots (**Figure 2E6**). Although the activity seems limited, *pStSTP1* can mediate a rather sink specific expression.

### Characterization of additional promoter sequences mediating higher tissue specificity

While some of the ten promoter elements tested during field trials could mediate a specific expression pattern for autotrophic tissues (e.g. *pSlRBCS2*, *pAtCAB1*) and some of them could mediate a rather specific expression pattern for heterotrophic organs (e.g. *pStSSS3*, *pStSTP1*), none of them appeared to be particularly strong and specific for the sink tissues. Therefore, we searched for additional promoter candidates in the literature or RNA sequencing datasets, with a particular focus on promoters with potential transport and heterotrophic storage tissues specificity and created additional reporter lines for 14 promoter-GUS constructs in an effort to identify a complete set of tissue-specific promoters for source-, transport- and sink tissues (**Table 2**).

**Table 2:** Summary of 14 promoters assayed via their GUS expression and/or GUS staining pattern in transgenic cassava plants grown in the greenhouse.

Name	Code	Source organism	Gene/Closest NCBI identifier	Reference	Expected tissue	Analyzed by	Results in Fig.
*CYTOSOLIC FRUCTOSE-1,6-BISPHOSPHATASE*	*StFBPase_cyt._*	*Solanum tuberosum*	*LOC102589275*	Ebneth, 1996	Source leaf mesophyll	Histology	S2
*PHOTOSYSTEM II SUBUNIT R*	*MePsbR*	*Manihot esculenta*	*Manes.15G102500*	This study	Autotrophic tissues	Histology + qPCR	4
*BIDIRECTIONAL SUGAR TRANSPORTER SWEET 1*	*MeSWEET1*	*Manihot esculenta*	*Manes.18G086400*	This study	Phloem and phloem parenchyma	Histology	8
*COMMELINA YELLOW MOTTLE VIRUS*	*CoYMV*	*Commelina yellow mottle virus*	*X52938.1*	Medberry et al., 1992	Phloem tissues	Histology	7
*GALACTINOL SYNTHASE 1*	*GolS1*	*Cucumis melo*	*AF249912.2*	Haritatos et al., 2000	Loading phloem	Histology	6
*SUCROSE SYNTHASE 1*	*MeSUS1*	*Manihot esculenta*	*Manes.03g044400*	This study	Phloem and phloem parenchyma	Histology + qPCR	9
*SUCROSE-PROTON SYMPORTER 2*	*AtSUC2*	*Arabidopsis thaliana*	*AT1G22710*	Truernit and Sauer, 1995	Phloem companion cells	Histology	5
B33 GENE	*StB33*	*Solanum tuberosum*	*X14483.1*	Rocha-Sosa et al., 1989	Heterotrophic tissues	Histology + qPCR	11
*DISCORIN 3 SMALL SUBUNIT*	*DjDio3*	*Discorea japonica*	*GU324672.1*	Arango et al., 2010	Heterotrophic tissues	Histology	S3
*GLUCOSE-6-PHOSPHATE/PHOSPHATE TRANSLOCATOR*	*MeGPT*	*Manihot esculenta*	*Manes.16G010700*	This study	Heterotrophic tissues	Histology + qPCR	13
*GRANULE-BOUND STARCH SYNTHASE 1*	*StGBSS1*	*Solanum tuberosum*	*X58453.1*	Van der Steege et al., 1992	Heterotrophic tissues	Histology + qPCR	12
*PATATIN CLASS 1*	*StPat*	*Solanum tuberosum*	*GQ352473*	Bevan et al., 1986	Heterotrophic tissues	Histology + qPCR	10
*MADS-BOX PROTEIN SRD1*	*IbSRD1*	*Ipomoea batatas*	*ACN39597.1*	Noh et al., 2010; Noh et al., 2012	Cambium and metaxylem	Histology	14
*CAULIFLOWER MOSAIC VIRUS 35S*	*CaMV35S*	*Cauliflower mosaic virus*	*MT233541.1*	Engler et al., 2014	All tissues	Histology + qPCR	S2

Promoters expected to be specific for photosynthetic tissues are highlighted in light green, promoters expected to be specific for phloem tissues are highlighted in yellow, promoters expected to be specific for heterotrophic storage organs are highlighted in light red, promoters expected to be specific for dividing tissues are highlighted in light blue, and promoters expected to be active ubiquitously are highlighted in light grey.

### Identification of additional promoters specific for autotrophic tissues

Two additional promoter-GUS constructs with an expected specificity for autotrophic tissues were created, the promoter of the cytosolic fructose-1,6-bisophosphatase *StFBPase*_cyt_ (1716 bp) and the promoter of *MePsbr* (2019 bp) were chosen. The promoter of StFBPase_cyt_ was chosen due to its previously demonstrated specificity for leaf mesophyll cells (Ebneth (1996), patents EP0938569, US6229067) and the promoter of *MePsbR* was chosen due to the high and leaf specific transcript levels of *MePsbR* in an RNA sequencing dataset (Kuon et al., 2019).

In contrast to the expected mesophyll-specific staining pattern, *pStFBPase_cyt_* showed considerable staining in the phloem- and cambium areas of stems (**Figure S3D**) and storage roots (**Figure S3E**), in addition to staining in the mesophyll of source (**Figure S3A**) and sink leaves (**Figure S3B**).

**Figure 3:**
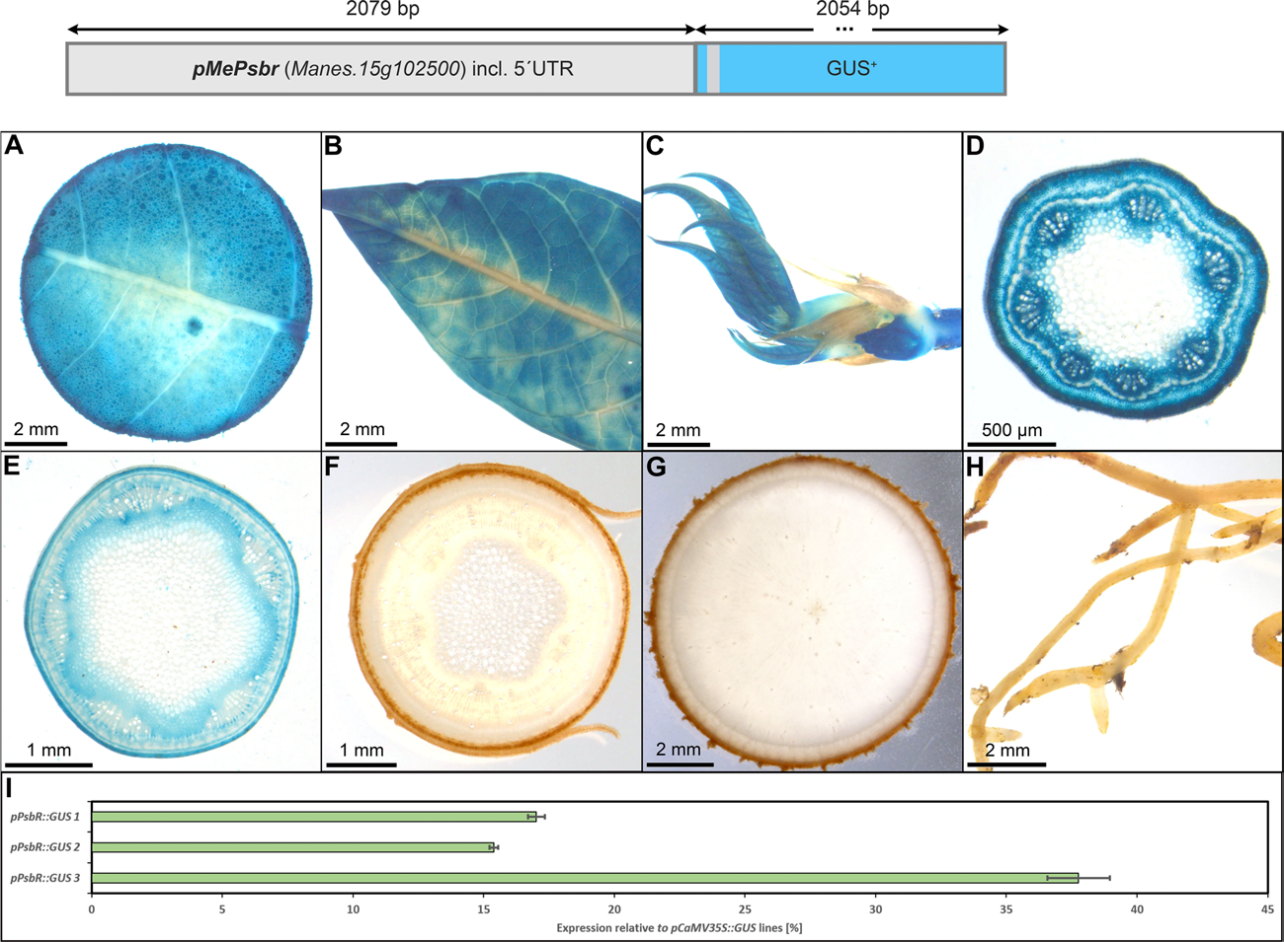
Representative GUS staining pattern of three *pMePsbr::GUS* promoter-reporter lines. **(A)** Source leaf, **(B)** Sink leaf, **(C)** Emerging leaves, **(D)** Petiole cross-section, **(E)** Upper stem cross-section, **(F)** Lower stem cross-section, **(G)** Storage root cross-section, **(H)** Fibrous roots, **(I)** GUS expression levels of three *pPsbR::GUS* lines relative to three *pCaMV35S::GUS* lines in %. Bars represent mean values with standard deviation (n=4).

However, a very specific staining pattern was found for *pMePsbR* (**Figure 3**). Here, staining was observed in the mesophyll of source leaves, sink leaves and newly emerging leaves (**Figures 3A–C**). Petiole cross-sections displayed labeling in most cell types beside sclerenchyma and pith tissue (**Figure 3D**) and upper stem sections displayed staining in the pith parenchyma, the phloem and cambium area, and the collenchyma (**Figure 3E**). The heterotrophic lower stem sections, storage roots and fibrous roots were completely devoid of GUS staining (**Figures 3F–H**). Therefore, the chosen promoter sequence for *MePsbR* can drive specific expression in autotrophic cassava tissues. To determine the approximate expression strength of *pMePbsbR*, we determined the relative expression level of the different lines and compared them to the relative expression levels of *pCaMV35* as determined in three *pCaMV35S::GUS* lines. The promoter of *MePsbR* showed approximately 15% to 35% activity compared to the promoter element of *CaMV35S* (**Figure 3I**). Similar expression levels were obtained for *pAtCAB1* and since *pCaMV35S* is a well-documented, strong promoter, the promoters of *MePsbR* can drive specific and reasonable strong expression in the autotrophic tissues of cassava.

### Identification of promoter sequences with predominant activity in phloem tissues

**Figure 4:**
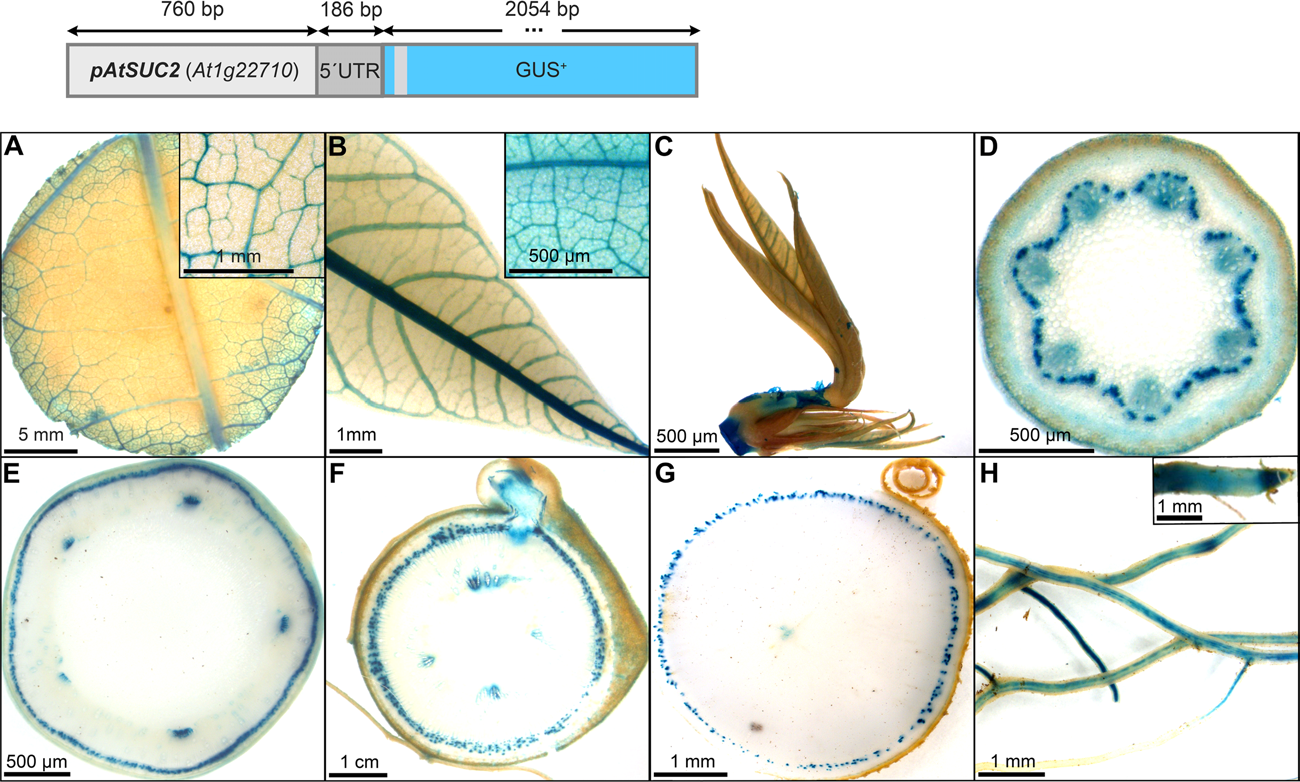
Representative GUS staining pattern of four *pAtSUC2::GUS* promoter-reporter lines. **(A)** Source leaf (Inlay = Close-up), **(B)** Sink leaf (Inlay = Close-up), **C** Emerging leaves, **(D)** Petiole cross-section, **(E)** Upper stem cross-section, **(F)** Lower stem cross-section, **(G)** Storage root cross-section, **(H)** Fibrous roots (Inlay = Root tip).

The promoter of *AtSUC2* (946 bp) was selected and expected to be phloem specific in cassava, since this promoter has been used as a phloem-specific tool in numerous studies in different species over the years [recently reviewed in Stadler and Sauer (2019)]. Indeed, *pAtSUC2::GUS* lines displayed pronounced staining in the minor and major veins of source leaves (**Figure 4A**), sink leaves (**Figure 4B**), newly developing leaves (**Figure 4C**), as well as a dotted staining in the phloem area of petioles (**Figure 4D**), upper stem (**Figure 4E**), lower stem (**Figure 4F**), and storage roots (**Figure 4G**). The dotted GUS staining in the phloem is very likely resulting from the staining of phloem companion cells. The vasculature of fibrous roots and the root tips also displayed GUS staining (**Figure 4H**). In addition, some staining was observed in protoxylem and xylem parenchyma areas (**Figure 4D, F**). These results demonstrate that *pAtSUC2* is well-suited to drive phloem companion cell specific expression also in cassava.

Two additional, well-known phloem promoters were chosen for testing in cassava: A 3000 bp-long promoter sequence of *Cucumis melo* driving expression of the *GALACTINOL SYNTHASE1* [*pGolS1*; Haritatos et al. (2000)] and a 1040 bp-long sequence from *Commelina Yellow Mottle Virus* (Medberry et al., 1992). The former sequence was previously described to have specific activity for the loading phloem, since GUS staining was specifically observed in the smallest veins of the source leaves (Haritatos et al., 2000). The later promoter sequence was described as a promoter with high-level expression, specific to phloem cells, as well as phloem-associated cells (Medberry et al., 1992). In addition, GUS staining was seen in phloem unloading tissues, like the tapetum (Medberry et al., 1992).

**Figure 5:**
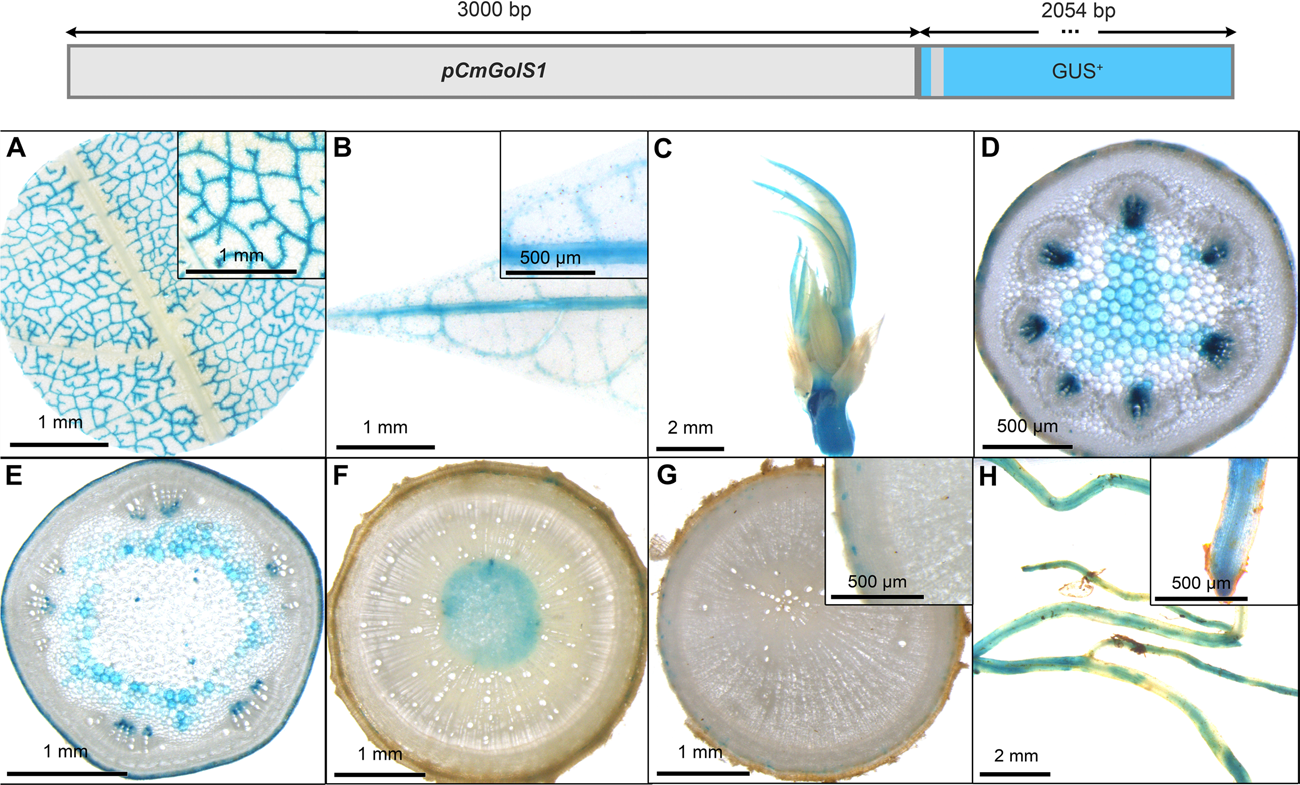
Representative GUS staining pattern of four *pCmGolS1* promoter-reporter lines. **(A)** Source leaf (Inlay = Close-up), **(B)** Sink leaf (Inlay = Close-up), **(C)** Emerging leaves, **(D)** Petiole cross-section, **(E)** Upper stem cross-section, **(F)** Lower stem cross-section, **(G)** Storage root cross-section (Inlay = Close-up), **(H)** Fibrous roots (Inlay = Root tip).

GUS staining of transgenic *pCmGolS1::GUS* plant lines revealed specific staining of minor veins in the source leaves in cassava (**Figure 5A**), matching the results obtained in previous publications (Haritatos et al., 2000). The majority of lines also displayed slightly patchy staining in the veins of sink and newly emerging leaves (**Figures 5B, C**), staining in the protoxylem/xylem parenchyma of petioles (**Figure 5D**) and green stems (**Figure 5E**), as well as slight staining in the pith tissue of auto- and heterotrophic stem tissue (**Figures 5E, F**). While storage roots displayed very little staining (**Figure 5G**), fibrous roots also displayed a slightly patchy staining (**Figure 5H**). Overall, the promoter sequence used, seemed mostly active in minor veins of source leaves but also seemed to convey some activity in non-phloem-related tissues in cassava. Despite the activity outside the leaf, the promoter could still be an interesting tool for biotechnological approaches centered on phloem loading.

**Figure 6:**
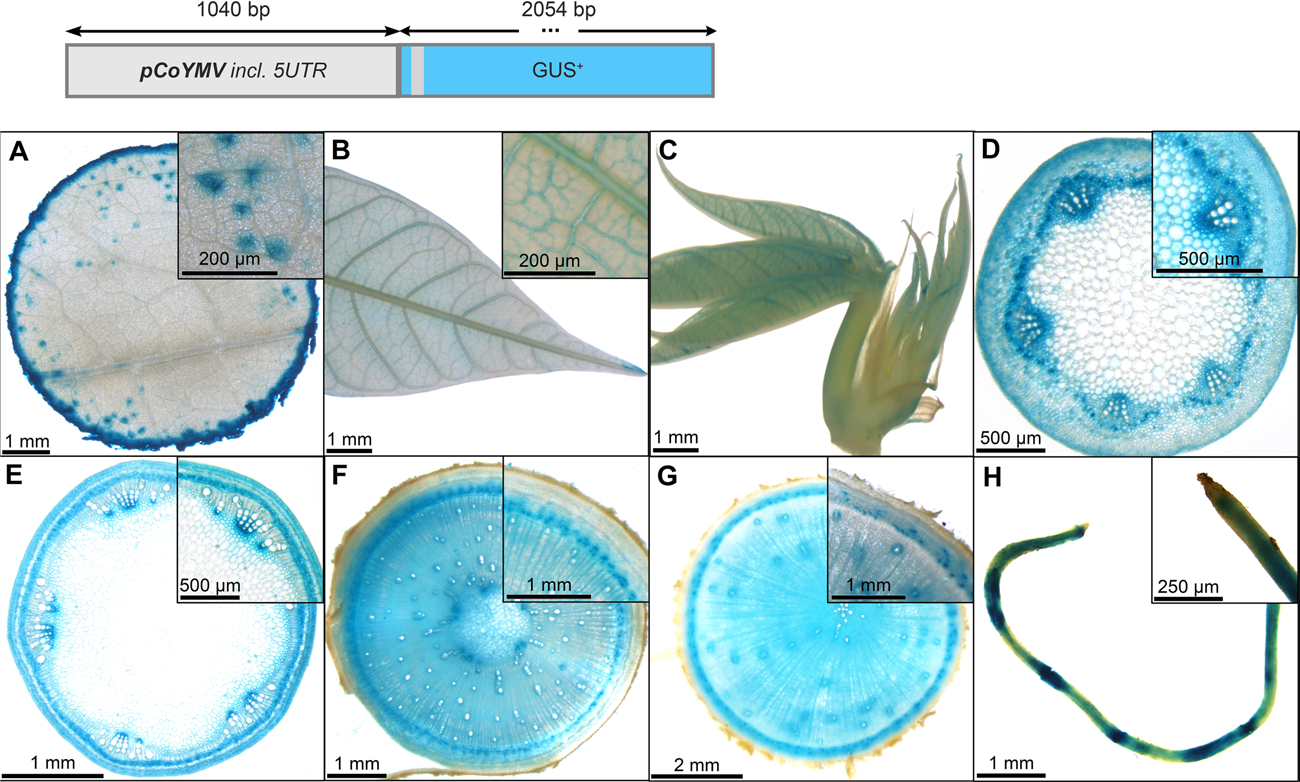
Representative GUS staining pattern of four *pCoYMV* promoter-reporter lines. **(A)** Source leaf (Inlay = Close-up), **(B)** Sink leaf (Inlay = Close-up), **(C)** Emerging leaves, **(D)** Petiole cross-section (Inlay = Close-up), **(E)** Upper stem cross-section (Inlay = Close-up), **(F)** Lower stem cross-section (Inlay = Close-up), **(G)** Storage root cross-section (Inlay = Close-up), **(H)** Fibrous roots (Inlay = Root tip).

In contrast to the *pCmGolS1::GUS* plant lines, which showed preferential activity in the loading phloem, the *pCoYMV::GUS* plant lines seemed to be more specific toward the transport- and unloading phloem. All lines studied, did not show any staining of source leaf vasculature, but rather displayed a staining pattern that seemed wound induced, due to the staining of the cutting site, as well as the punctual staining within the mesophyll (**Figure 6A**) or in fibrous roots (**Figure 6H**). In the sink leaves, the staining was observed just outside the vasculature, potentially representing the phloem parenchyma (**Figures 6B, C**). In addition to some staining in protoxylem and pith parenchyma (**Figures 6D, F**), pronounced staining was observed in the phloem tissues of petioles (**Figure 6D**), autotrophic stems (**Figure 6E**), heterotrophic stems (**Figure 6F**), and storage roots (**Figure 6G**). Interestingly, tissues with important functions in lateral transport, as indicated by the staining of vascular rays in the lower stems and storage roots, were also stained in these promoter-reporter plants (**Figures 6F, G**). Taken together, the analyzed sequence of *pCoYMV* seemed rather specific towards transport and unloading phloem tissues, which is in line with previous results, showing promoter activity in vascular and reproductive tissues (Medberry et al., 1992). Although not a quantitative measure, all *pCoYMV::GUS* lines stained within seconds of adding staining buffer, indicating a very strong activity for transport and unloading phloem tissues.

**Figure 7:**
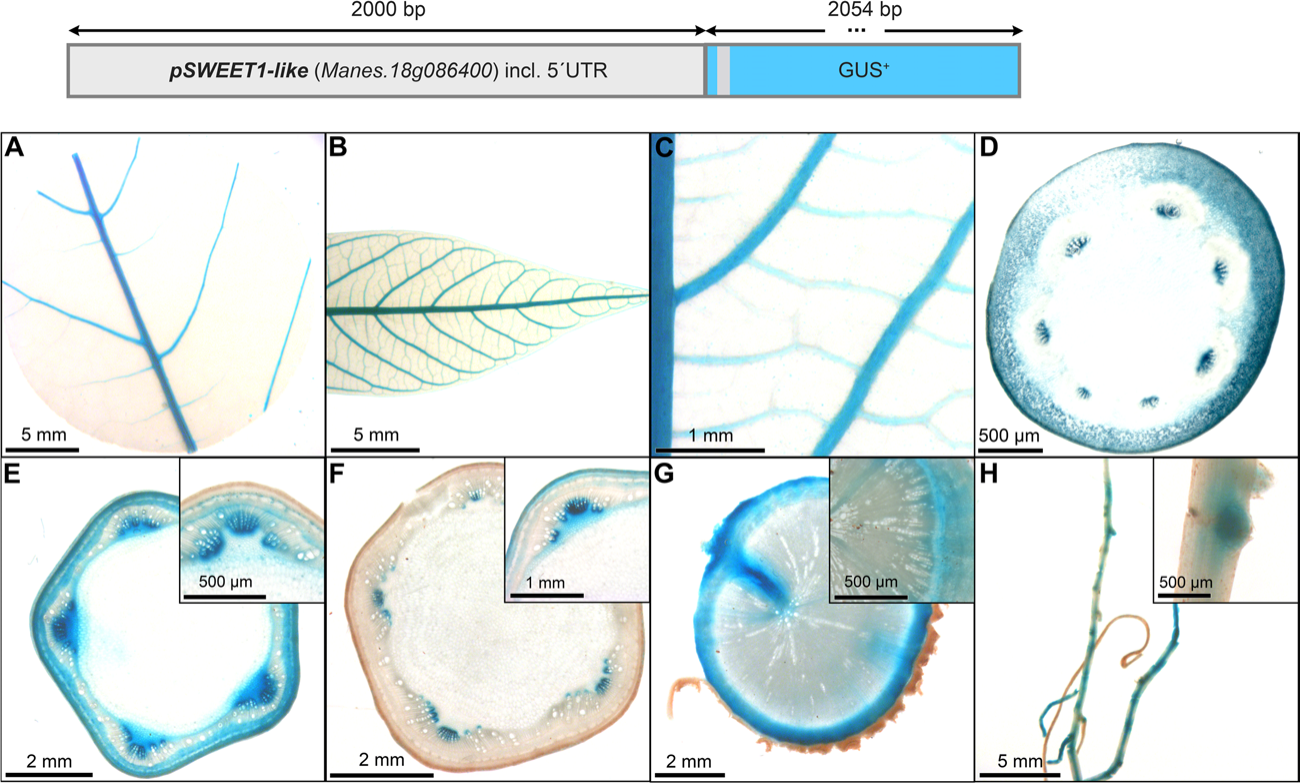
Representative GUS staining pattern of at least four *pMeSWEET1-like* promoter-reporter lines. **(A)** Source leaf, **(B)** Sink leaf, **(C)** Emerging leaves, **(D)** Petiole cross-section, **(E)** Upper stem cross-section (Inlay = Close-up), **(F)** Lower stem cross-section (Inlay = Close-up), **(G)** Storage root cross-section (Inlay = Close-up), **(H)** Fibrous roots (Inlay = Developing side root).

The promoter of *pMeSWEET1-like* (**Figure 7**) also displayed staining in the phloem areas, although less specific compared to p*AtSUC2* (**Figure 4**). The promoter element of *MeSWEET1-like* (2000 bp) was initially selected for testing because its transcript appeared highly abundant in storage roots in a RNA-seq dataset (NCBI BioProject ID PRJNA784380). Promoter-GUS lines revealed staining in the vasculature of source- and sink leaves (**Figures 7A, B**), as well as staining in phloem and parenchyma tissues of petioles and stems (**Figures 7D–G**). The outer storage root region, containing phloem and phloem parenchyma, displayed pronounced GUS staining (**Figure 7G**). In addition, *pMeSWEET1-like* showed activity in the fibrous root vasculature and root tips (**Figure 7H**). These results indicate that *pMeSWEET1-like* has preferential activity in phloem and parenchyma cells in cassava.

**Figure 8:**
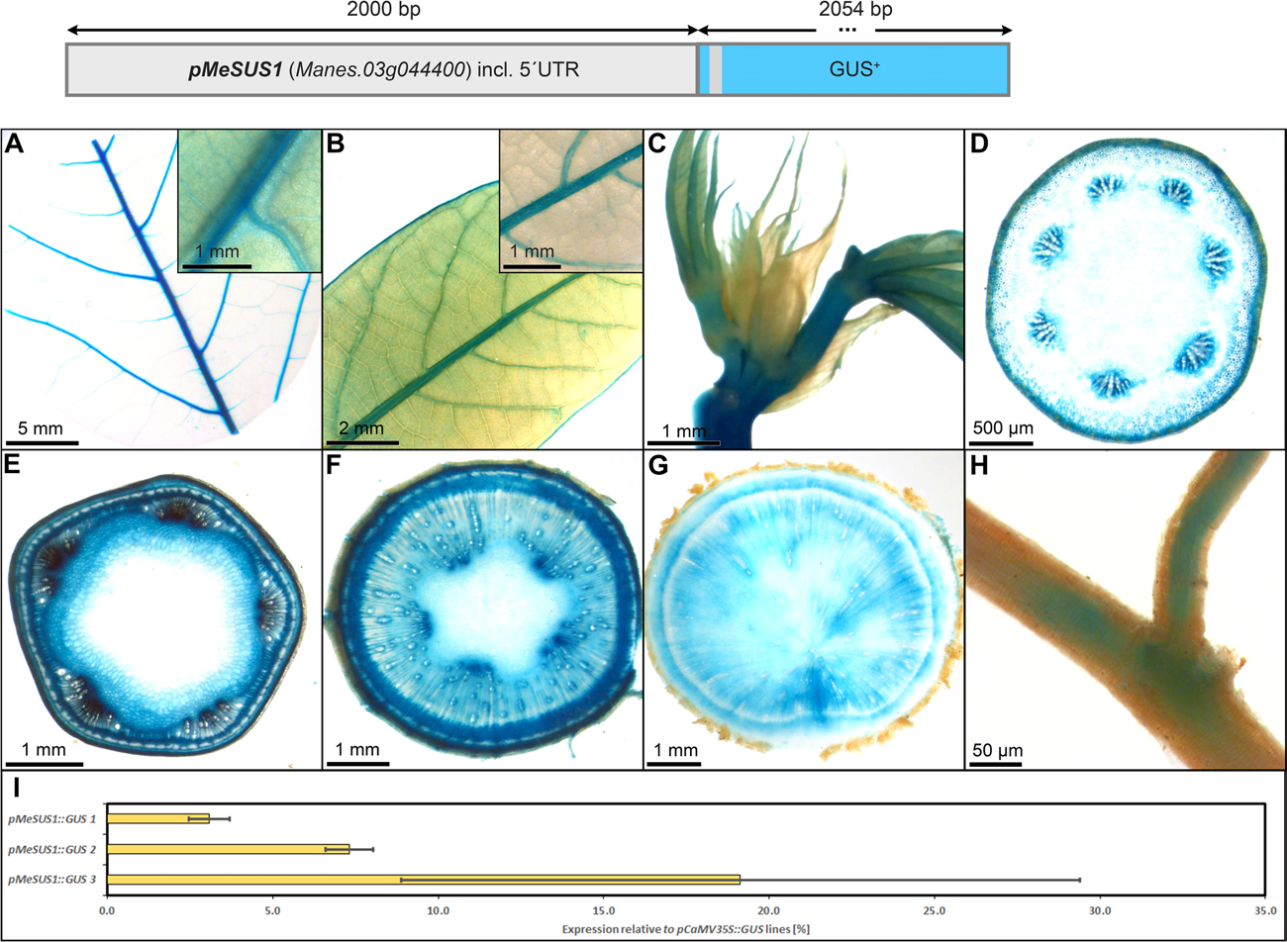
Representative GUS staining pattern of at least four *pMeSUS1* promoter-reporter lines. **(A)** Source leaf (Inlay = Close-up), **(B)** Sink leaf (Inlay = Close-up), **(C)** Emerging leaves, **(D)** Petiole cross-section, **(E)** Upper stem cross-section, **(F)** Lower stem cross-section, **(G)** Storage root cross-section, **(H)** Fibrous roots, **(I)** GUS expression levels of three *pMeSUS1::GUS* lines relative to three *pCaMV35S::GUS* lines in %. Bars represent mean values with standard deviation (n=4).

The promoter of *MeSUS1* (2000 bp) was chosen for testing as a putative phloem promoter because *MeSUS1* transcripts were found to be highly abundant in the phloem fraction of cassava storage root tissues in a RNA-seq datasets (NCBI BioProject ID PRJNA784380). The *pSUS1::GUS* lines displayed an interesting staining pattern, resembling the *pCoYMV* promoter (**Figure 6**). *pSUS1* was active in the major veins of the leaf vasculature (**Figures 8A, B**), in the shoot apex (**Figure 8C**), in phloem and parenchyma cell types (**Figures 8D–G**), and in fibrous root vasculature (**Figure 8H**). It displayed pronounced staining in vascular rays of stems and storage roots (**Figures 8F, G**) and the staining pattern in the storage roots indicated preferential activity in the phloem unloading area, as well as in young xylem cells of the storage roots (**Figure 8G**). This staining pattern matches the previously described symplasmic unloading mode of cassava and the previously observed metabolic gradients within the storage root (Mehdi et al., 2019).

To determine the approximate expression strength of *pMeSUS1*, we tested the relative expression level of the different lines and compared them to the relative expression levels of *pCaMV35* as determined in three *pCaMV35S::GUS* lines. The promoter elements of *MeSUS1* showed approximately 5-20% activity compared to the promoter element of *CaMV35S* (**Figure 8I**). This is considerably weaker as the expression strength of the more parenchyma-dominated promoters shown below. However, the promoter is active in far less cells across the storage root, thinning out the specific signal. Overall, *pMeSUS1* is an interesting option as a promoter for applications focused on phloem transport and unloading.

### Identification of promoter sequences with predominant activity in heterotrophic storage tissues

Storage root specific promoters are of special interest for cassava because they enable the modification of agronomically interesting storage root traits like starch content, starch quality, nutritional improvements, or shelf life. To our knowledge, there is only one storage root-specific promoter, which was been tested and confirmed in cassava by independent groups. A particular promoter sequence of the potato *Patatin Class I* promoter [*pStPat*; Bevan et al. (1986)], coding for the tuber storage protein patatin, mediates this specific expression pattern in cassava [e.g. Ihemere et al. (2006); Zidenga et al. (2012); Vanderschuren et al. (2014); Gaitan-Solis et al. (2015); Li et al. (2015); Zhou et al. (2017); Beyene et al. (2018); Wang et al. (2018); Narayanan et al. (2019)].

The promoter element of *pStPat* (999 bp) was included in this study to get confirmation of its tissue specificity and activity. In addition, the promoter elements of *pStB33* (1529 bp), *pStGBSS1* (1061 bp), *pDjDIO3* (1925 bp), and *pMeGPT* (2000 bp) were selected for testing and assumed to be preferentially active in starch storage tissues. The promoters of *StB33*, *StGBSS1*, and *DjDIO3* were previously published with preferential storage organ activity in other plants (Rocha-Sosa et al., 1989; Van der Steege et al., 1992; Arango et al., 2010). The promoter of *MeGPT* was chosen, because *MeGPT* transcripts were found highly abundant in storage root tissues in prior RNA-seq datasets (NCBI BioProject ID PRJNA784380) and where found to accumulate during storage root bulking (Rüscher et al., 2021).

**Figure 9:**
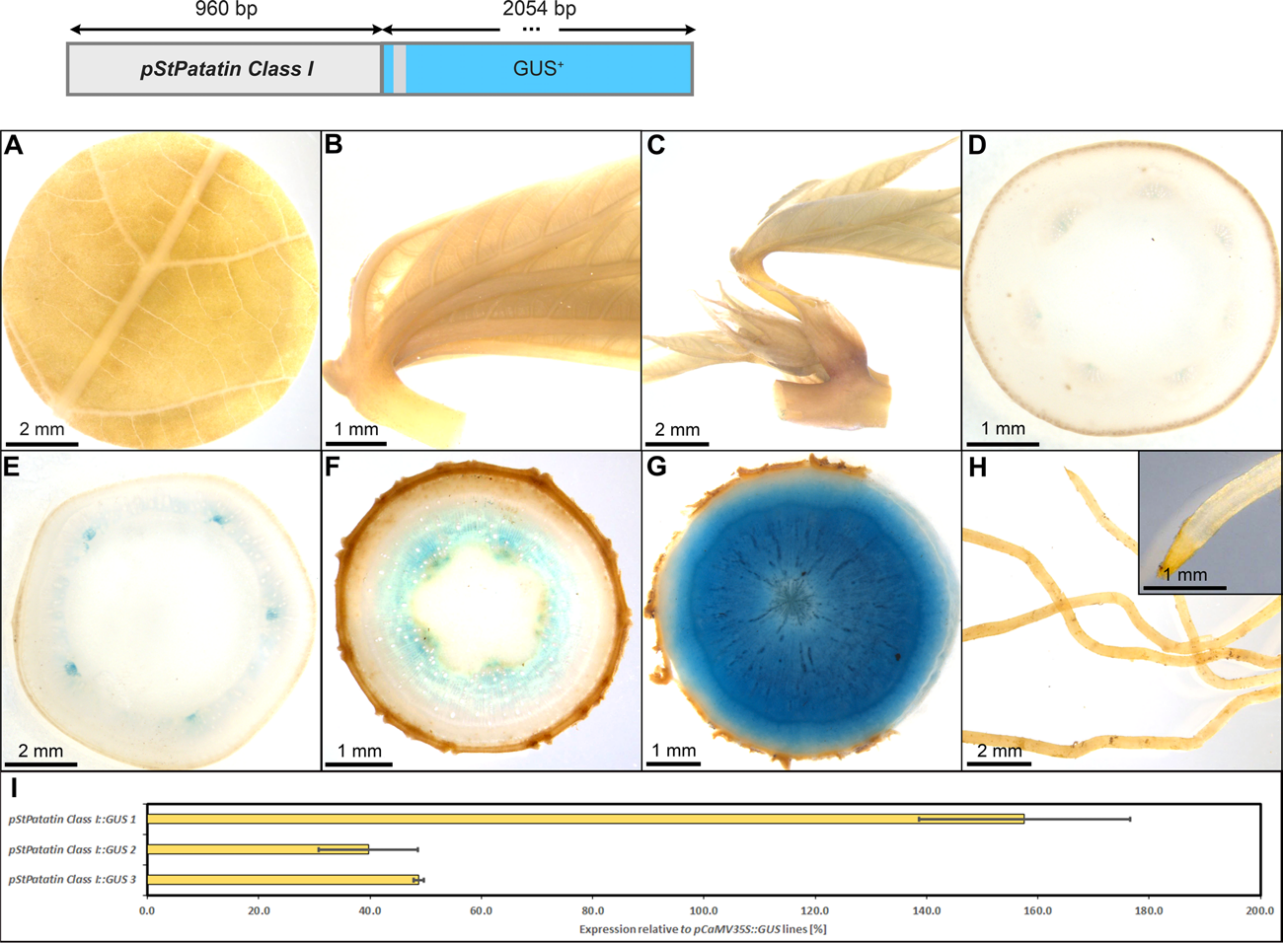
Representative GUS staining pattern of four *pStPatatin Class I* promoter-reporter lines. **(A)** Source leaf, **(B)** Sink leaf, **(C)** Emerging leaves, **(D)** Petiole cross-section, **(E)** Upper stem cross-section, **(F)** Lower stem cross-section, **(G)** Storage root cross-section, **(H)** Fibrous roots, **(I)** GUS expression levels of three *pStPatatin : GUS* lines relative to three *pCaMV35S::GUS* lines in %. Bars represent mean values with standard deviation (n=4).

As expected, *pStPat* displayed strong expression in storage roots, as well as the highest specificity for storage root expression among all promoters tested. The lines displayed no staining in leaves and petioles (**Figures 9A–D**), only faint staining in upper and lower stems (**Figures 9E, F**), as well as no staining in fibrous roots (**Figure 9H**). However, strong staining was observed in the xylem core area of the storage root (**Figure 9G**), consisting mostly of xylem parenchyma cells. The relative expression level of *pStPat*, compared to the relative expression level of *pCaMV35*, was approximately 40-160%, depending on the respective line (**Figure 9I**). These results underscore the storage root specificity of *pStPat* in cassava and confirm a high promoter activity in storage roots.

**Figure 10:**
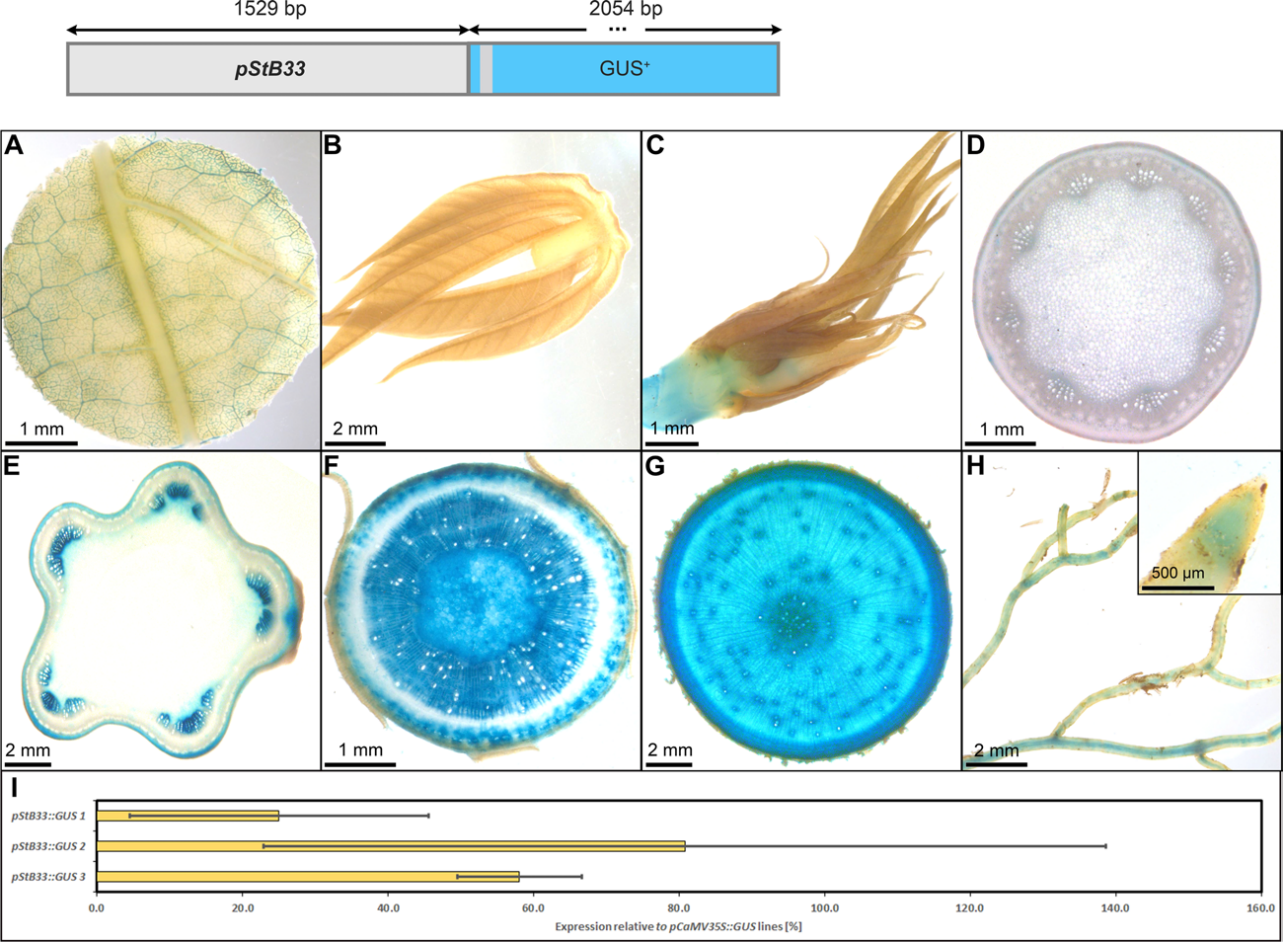
Representative GUS staining pattern of at least four *pStB33* promoter-reporter lines. **(A)** Source leaf, **(B)** Sink leaf, **(C)** Emerging leaves, **(D)** Petiole cross-section, **(E)** Upper stem cross-section, **(F)** Lower stem cross-section, **(G)** Storage root cross-section, **(H)** Fibrous roots (Inlay = Root tip), **(I)** GUS expression levels of three *pStB33::GUS* lines relative to three *pCaMV35S::GUS* lines in %. Bars represent mean values with standard deviation (n=4).

The promoter of *StB33*, also part of the class I family of patatin genes (Rocha-Sosa et al., 1989), appeared very suitable to drive strong expression in heterotrophic storage tissues in cassava as well. The p*StB33::GUS* lines, displayed staining of minor veins in source leaves and no staining in sink leaves and petioles (**Figures 10A–D**). Upper stem tissue showed staining of collenchyma and protoxylem (**Figure 10E**), while the heterotrophic lower stem section (**Figure 10F**) and storage roots displayed strong staining in xylem and phloem parenchyma (**Figure 10G**). In addition, the vasculature and root tips of fibrous roots were stained (**Figure 10F**). The relative expression level of *pStB33*, compared to the relative expression level of *pCaMV35*, was approximately 20-80%, depending on the respective line. Therefore, *pStB33* is rather specific for sink tissues and has a high activity in sink organs, although the activity might be slightly lower compared to the *StPat* promoter.

**Figure 11:**
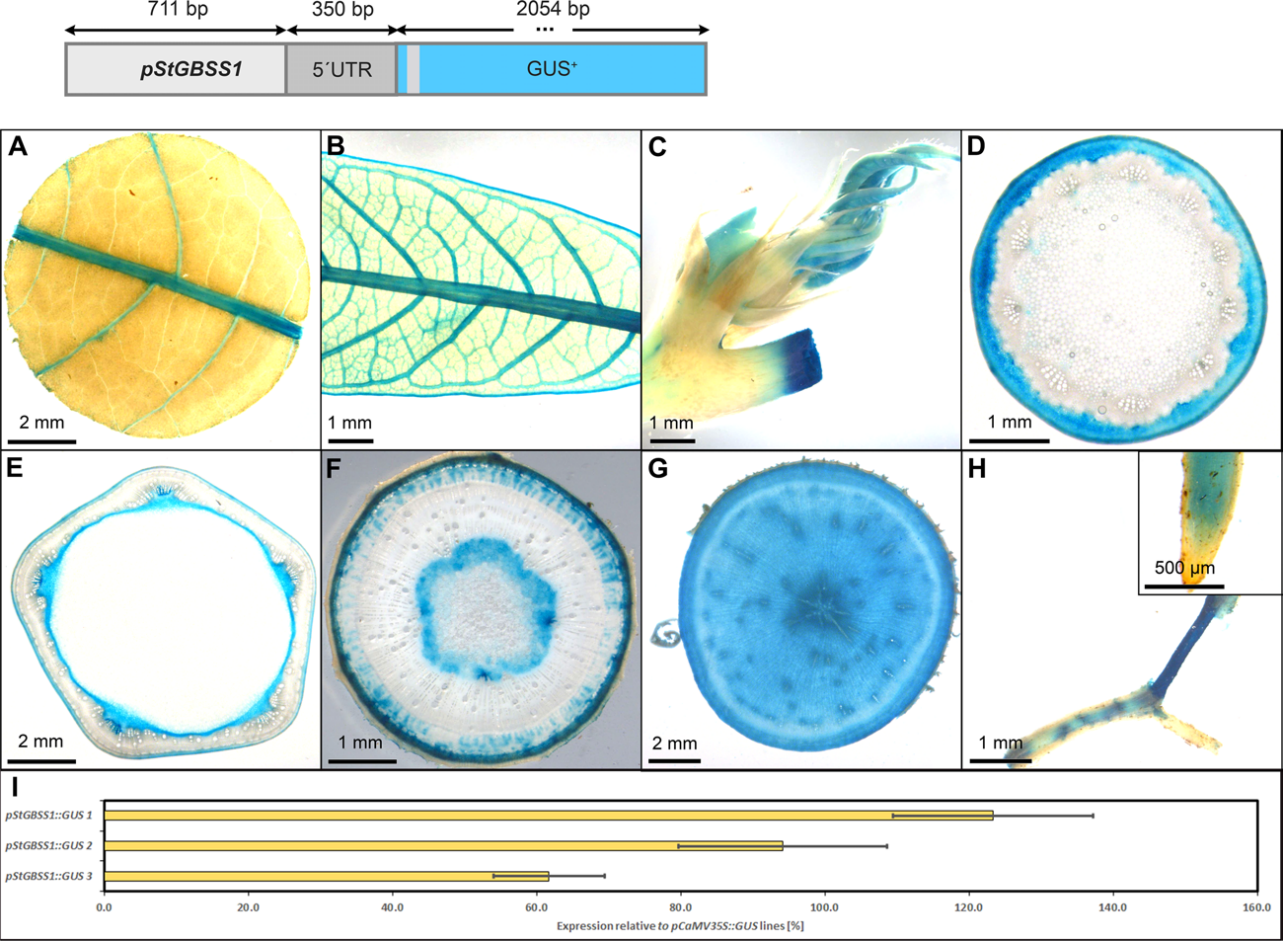
Representative GUS staining pattern of four *pStGBSS1* promoter-reporter lines. **(A)** Source leaf, **(B)** Sink leaf, **(C)** Emerging leaves, **(D)** Petiole cross-section, **(E)** Upper stem cross-section, **(F)** Lower stem cross-section, **(G)** Storage root cross-section, **(H)** Fibrous roots (Inlay = Root tip), **(I)** GUS expression levels of three *pStGBSS1:GUS* lines relative to three *pCaMV35S::GUS* lines in %. Bars represent mean values with standard deviation (n=4).

The p*StGBSS1::GUS* lines displayed a staining pattern with predominant activity in the phloem- and xylem parenchyma cells of storage roots (**Figure 11G**). They also displayed staining in the shoot apex (**Figure 11C**), in the collenchyma of petioles and stems (**Figures 11D–F**), the pith parenchyma (**Figure 11E, F**), and the vasculature of fibrous roots (**Figure 11H**). In contrast to the two patatin promoters *pStPat* and *pStB33* (**Figures 9**, **10**), *pStGBSS1* showed activity in both source- and sink leaf vasculature (**Figures 11A, B**). The relative GUS expression level caused by *pStGBSS1*, compared to the relative GUS expression level caused by *pCaMV35*, was approximately 60-120%, depending on the respective line (**Figure 11I**). Therefore, *pStGBSS1* displays a similar sink activity as *pStPat*, but seems less specific due to its higher activity in some cell types of leaves, petioles and stems.

**Figure 12:**
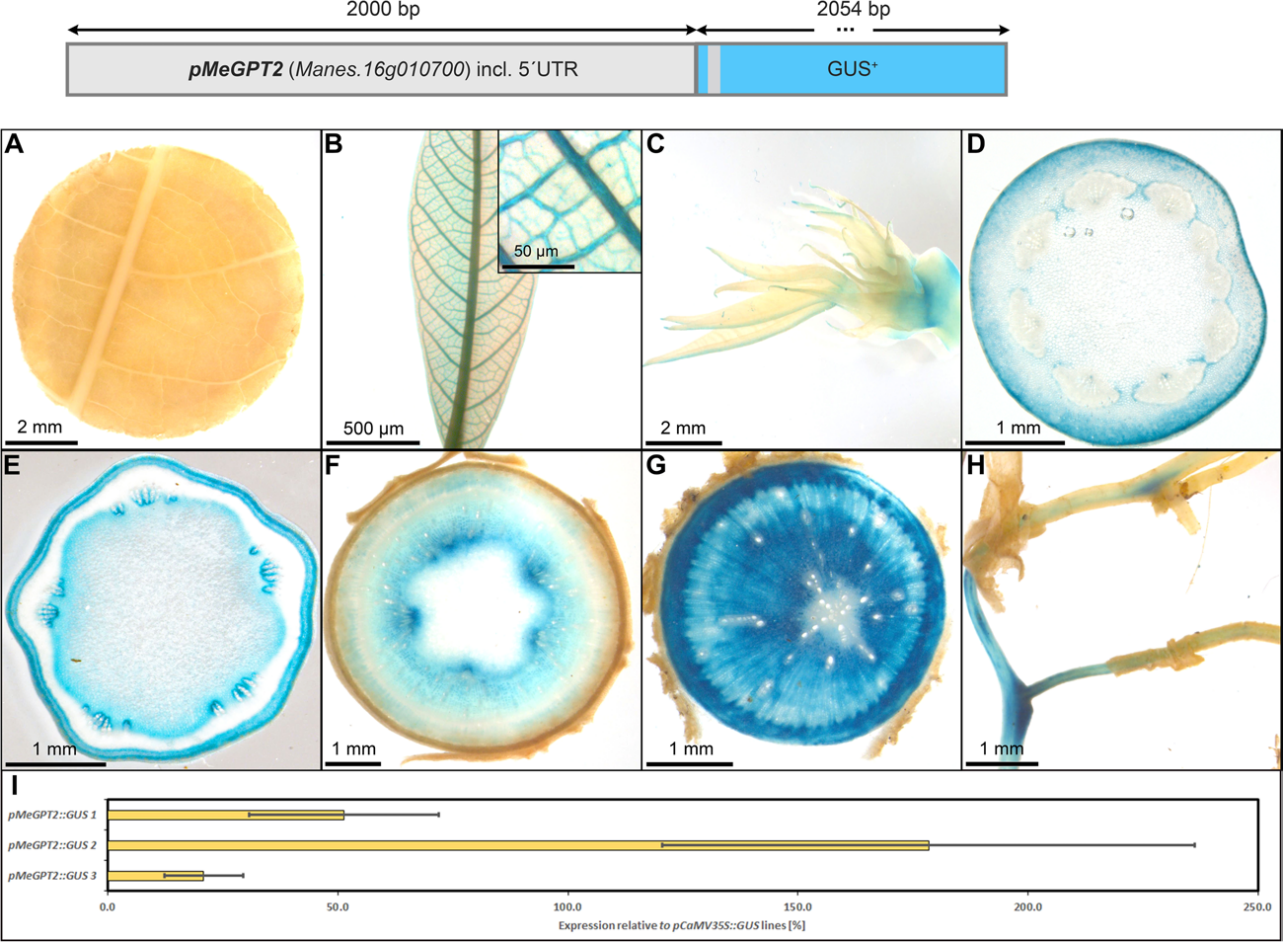
Representative GUS staining pattern of four *pMeGPT2* promoter-reporter lines. **(A)** Source leaf, **(B)** Sink leaf (Inlay = Close-up), **(C)** Emerging leaves, **(D)** Petiole cross-section, **(E)** Upper stem cross-section, **(F)** Lower stem cross-section, **(G)** Storage root cross-section, **(H)** Fibrous roots, **(I)** GUS expression levels of three *pMeGPT2::GUS* lines relative to three *pCaMV35S::GUS* lines in %. Bars represent mean values with standard deviation (n=4).

The promoter of *MeGPT* showed a similar staining pattern compared to *pStGBSS1*, with predominant activity in the phloem- and xylem parenchyma cells of storage roots (**Figure 12G**). It also displayed staining in the shoot apex (**Figure 12C**), in the collenchyma of petioles and stems (**Figures 12D–F**), the pith parenchyma (**Figures 12E, F**), and the vasculature of fibrous roots (**Figure 12H**). However, it had no staining in source leaves (**Figure 12A**) and only staining in sink leaf vasculature (**Figure 12B**). The relative GUS expression level caused by *pMeGPT*, compared to the relative GUS expression level caused by *pCaMV35*, was approximately 20-150%, depending on the respective line (**Figure 12I**). Taken together, the promoter of *MeGPT* appears rather specific for heterotrophic storage tissues and displays activity in the same range as the *StPat* promoter.

While *pStPat*, *pStB33*, *pStGBSS1*, and *pMeGPT* all show preferential activity in heterotrophic storage tissues, the promoter of the *dioscorin 3 small subunit* gene from *Discorea japonica (DjDIO3*) did not. In contrast to what was previously suggested by Arango et al. (2010), *pDjDIO3::GUS* lines displayed a rather ubiquitous staining pattern in cassava (**Figure S4**).

### Identification of a promoter sequence with predominant activity in cambial tissues

To realize transgenic interventions targeting cassava secondary growth, promoters with distinct activity in the vascular cambium could be useful tools. We tested the tissue specificity of the sweet potato MADS-box transcription factor *pIbSRD1* (3011 bp) in cassava, a promoter that was previously characterized in thale cress, carrot, potato and sweet potato. In sweet potato, the *SRD1* expression was shown to be auxin-responsive and the transcript was localized in the primary cambium, secondary cambium, and primary phloem cells (Noh et al., 2010). The main promoter activity in thale cress could be demonstrated in the vasculature including pericycle and endodermis, while the promoter activity was strong in all cells of carrot taproots and potato tubers (Noh et al., 2012).

**Figure 13:**
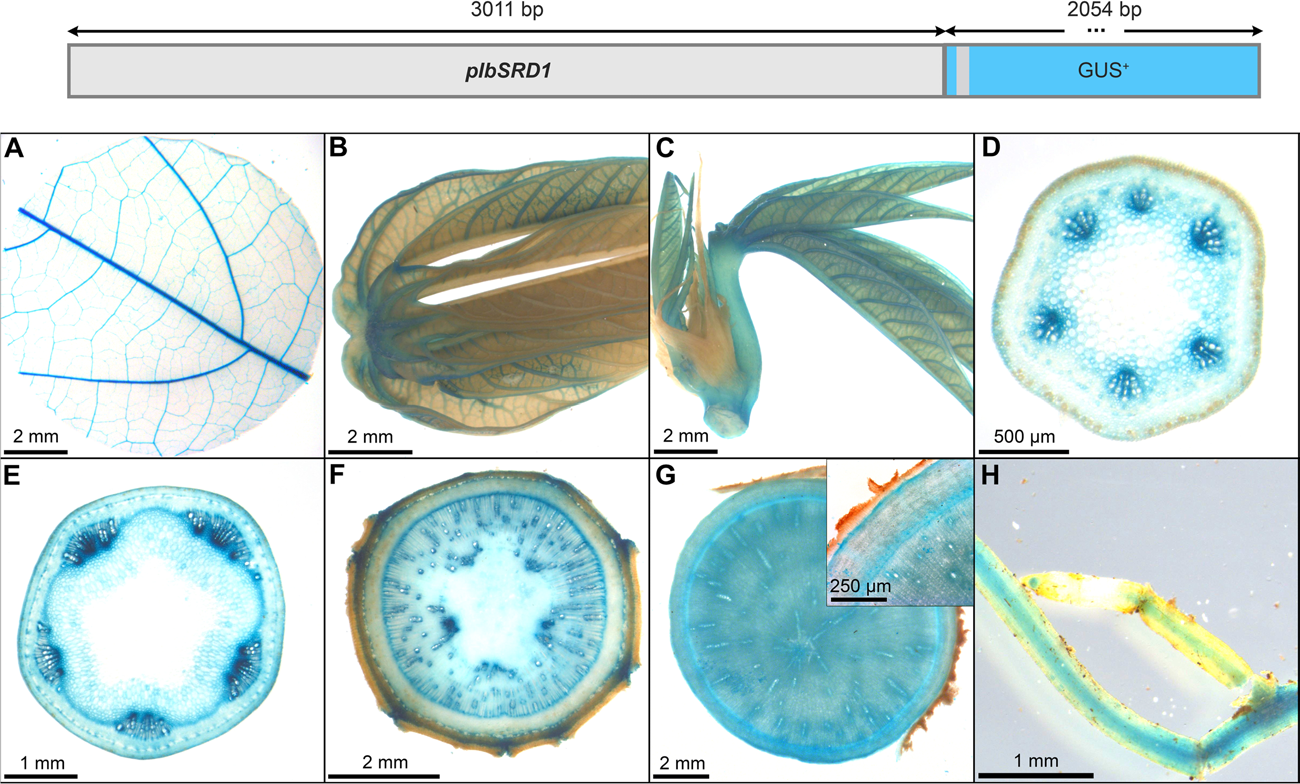
Representative GUS staining pattern of at least four *pIbSRD1* promoter-reporter lines. **(A)** Source leaf, **(B)** Sink leaf, **(C)** Emerging leaves, **(D)** Petiole cross-section, **(E)** Upper stem cross-section, **(F)** Lower stem cross-section, **(G)** Storage root cross-section (Inlay = Close-up), **(H)** Fibrous roots.

The promoter activity in cassava resembles the results obtained for sweet potato and thale cress. Pronounced staining was observed in the vasculature of source leaves, sink leaves, newly emerging leaves (**Figures 13A–C**), and the vasculature of fibrous roots (**Figure 13H**), as well as in the protoxylem and xylem vessels of petiols and stems (**Figures 13D, E**). In addition, strong staining was observed in the vascular cambium and cork cambium of stems and storage roots (**Figures 13E-G**). Together these results demonstrate that *pIbSRD1* has specific activity for cells with meristematic identity in cassava.

### Summary of observed promoter specificities

Among the tested leaf promoters, *StFBPase_cyt_*, *AtFBA2*, *AtGAPA*, *StLS1*, and *AtRBCS3B* displayed an either weak or unspecific expression. However, the promoters of *AtCAB1* and *MePsbR* proved specific and reasonably strong, making them well-suited tools for transgene expression in photosynthetic tissues of cassava. Although no dedicated promoter-GUS lines were created for the promoters of *SlRBCS2* and *AtRBCS1A*, they appeared very active in source leaf tissues in transcript studies. In addition, *pSlRBCS2* also appeared to be specific for this tissue.

The tested promoters of *AtSUC2*, *CmGolS1*, *CoYMV*, *MeSWEET1-like*, and *StSTP1* can be used as expression tools for phloem tissues. While *pAtSUC2* has specific expression along the entire phloem, *pCmGolS1* or *pCoYMV* can target the loading or transport/unloading phloem, respectively. The promoter of *MeSWEET1-like and StSTP1* can be used to target phloem and especially phloem parenchyma tissues of cassava. The promoter of *MeSUS1* also has considerable phloem activity, as well as storage tissue activity, especially in the cells closer to the vascular cambium. This sequence could be an interesting tool for approaches centered on increased sink demand.

Among the promoters with predominant activity in heterotrophic storage tissue, *MeGBSS1* and *StSSS3* seemed less suitable promoters due to their low specificity, or in the case of *pStSSS3* weak activity. The promoter of *StPatatin Class I* proved to be very active and very storage root specific, as previously described. However, *pStB33*, *pMeGPT2* and *pStGBSS1* are also very good promoters for sink tissue expression, as they are predominantly active in starch-storing stem and storage root tissues. They also seem to have a comparable expression strength compared to *pStPatatin Class I*. These promoters will be useful to realize larger transgene stacks that try to avoid repetition of the same promoter sequence in order to avoid silencing or recombination effects.

While *pDjDIO3* is likely very strong (as it showed a strong GUS staining within seconds of staining buffer addition), the promoter is very unspecific and there is a large number of options for this expression pattern. In contrast, *pIbSRD1* showed a highly specific expression pattern with high activity in dividing cells. This promoter can be an interesting tool for more developmental focused approaches targeting stem cells.

Taken together, we have confirmed a number of tissue-specific promoter elements, allowing targeted transgene expression in a variety of cassava tissues. We summarize our recommendations for the most specific promoters per tissue in **Table 3**. We hope that these promoter sequences will support further transgenic studies in cassava and prove useful for the cassava community.

**Table 3:** Promoter recommendations for target tissues.

Name	Code	Confirmed tissue specificity	Results in Fig.
*CHLOROPHYLL A/B-BINDING PROTEIN*	*AtCAB1*	Autotrophic tissues	3
*PHOTOSYSTEM II SUBUNIT R*	*MePsbR*	Autotrophic tissues	4
*RIBULOSE BISPHOSPHATE CARBOXYYLASE SMALL SUBUNIT 2*	*SlRBCS2*	Autotrophic tissues	1
*BIDIRECTIONAL SUGAR TRANSPORTER SWEET1*	*MeSWEET1*	Phloem and phloem parenchyma	8
*SUCROSE-PROTON SYMPORTER 2*	*AtSUC2*	Phloem companion cells	5
*SUCROSE SYNTHASE 1*	*MeSUS1*	Phloem and parenchyma cells	9
*B33 GENE*	*StB33*	Heterotrophic tissues	11
*GLUCOSE-6-PHOSPHATE/PHOSPHATE TRANSLOCATOR*	*MeGPT*	Heterotrophic tissues	13
*GRANULE-BOUND STARCH SYNTHASE1*	*StGBSS1*	Heterotrophic tissues	12
*PATATIN CLASS 1*	*StPat*	Heterotrophic tissues	10
*MADS-BOX PROTEIN SRD1*	*IbSRD1*	Cambium and metaxylem	14

Promoters observed to be specific for photosynthetic tissues are highlighted in light green, promoters observed to be specific for phloem tissues are highlighted in yellow, promoters observed to be specific for heterotrophic storage organs are highlighted in light red, and promoters observed to be specific for dividing tissues are highlighted in light blue.

## Discussion

Alongside cassava breeding, trait improvement for this important crop can be achieved through biotechnology by genome editing or transgene expression, introducing additional genetic variety or new functionalities. While traits like herbicide- or pathogen-resistance can sometimes be improved by transferring only a single gene, most traits, like significant nutritional improvements or even yield, often require the transfer and expression of multiple genes. In addition, it is desirable to combine different transgenic traits to aim for plants that are resistant to biotic and abiotic stress, high yielding and nutritious. Subsequent breeding in target genotypes is facilitated by linked transgenes, i.e. transgenes that have integrated into a particular genomic positon together.

However, expressing a variety of linked transgenes with particular strength and tissue specificity is a challenge and there are different ways of approaching it: Polycistronic- or polyprotein strategies have been developed, which can express multiple genes under the control of a single regulatory sequence by either combining all transgenes into a single transcript with subsequent individual translation, or by posttranslational cleavage of a long polypeptide chain, releasing the desired proteins [for review see Halpin (2005)]. However, both methods have limitations, especially if expression in different tissues or subcellular compartments is required. With recent advances in cloning strategies and falling prices for gene synthesis, as well as improvements to transformation protocols allowing for the transformation of larger pieces of DNA, multigene construct-based strategies have become favorable. In these constructs, the individual expression cassettes can be adjusted according to the desired subcellular localization and tissue specificity provided suitable promoters are available. There have been many reports, highlighting the potential of this strategy for i.e. nutritional or yield improvement in different plants (Ye et al., 2000; Paine et al., 2005; Jonik et al., 2012; Li et al., 2015; Kromdijk et al., 2016; Narayanan et al., 2019; South et al., 2019; Wu et al., 2019; Lopez-Calcagno et al., 2020; Narayanan et al., 2021).

This approach, however, requires the availability of a variety of promoters, especially if transgene expression in different tissues is desired. Reusing identical promoters to drive target gene expression in a particular tissue can work, but has the risk of causing recombination or transgene silencing effects, as reported already 30 years ago (Jorgensen, 1992). The existence of a variety of well-characterized promoters, with particular strength and specificity avoids these risks.

The promoter controlling the specificity and expression strength of a given transcript is always dependent on the particular sequence and sequence environment. For instance, combining multiple promoters in close proximity into multigene constructs might result in promoter crosstalk, altering promoter activity and/or specificity. Interestingly, we have observed a large overlap between the results for promoter specificity obtained from multigene constructs and the results obtained from individual promoter-gus plants, suggesting only limited crosstalk between the promoters in the multigene constructs. This observation supports the observed promoter specificities presented in this study and suggests that multiple transgenes can be simultaneously expressed in a tissue-specific manner through a multigene construct, provided suitable promoters are used.

However, our results underline that the described promoter activities and specificities from other plants are often not easily transferable to cassava, highlighting the current need for targeted promoter testing directly in cassava. Overall, we had more success isolating tissue-specific promoter sequences by relying on information from tissue-specific transcript datasets and testing endogenous promoters with a sequence length around 2000bp. Since tissue-specific expression is due to cell type-specific promoter activity, it is understandable that these endogenous promoters have a higher probability to contain the required cis elements for promoter activation in a particular cell type and/or have a higher probability to contain the necessary cis elements for suppression of promoter activity in other cell types. While the use of endogenous promoters seems to have a higher probability to achieve tissue-specific expression of the desired transgene, their activity also has a higher probability to be subject to endogenous regulation mechanisms. The use of the cassava´s own *GPT* promoter for instance would be beneficial to coordinate transgene expression with the onset of storage root formation, since the transcript greatly increases in expression during storage root bulking (Rüscher et al., 2021). At the same time, the likelihood of silencing at some point during cassava growth in response to certain environmental cues seems higher for the endogenous GPT promoter, compared to a potato-derived promoter like *PATATIN CLASS I* for instance.

Despite the higher likelihood of unexpected expression patterns while testing promoter sequences derived from other plants, sometimes exactly these unexpected findings are also the most interesting. Interestingly, almost all sink-specific promoters tested, including the potato *PATATIN B33* promoter, showed activity in tissues containing xylem parenchyma cells like the vasculature, the lower stem, and the storage root. By contrast, the potato *PATATIN CLASS I* promoter (approximately 70% sequence identity to the *PATATIN B33* promoter), which is also expected to be active in all xylem parenchyma cells, displayed a clearly higher specificity with almost exclusive activity in storage roots. Therefore, targeted testing of both endogenous and heterologous promoter sequences can yield highly useful expression tools for cassava research.

It would certainly be interesting for future studies to identify cell-type specific transcription factor regulatory elements for cassava promoters in an attempt to design artificial tissue-specific minimal promoters. However, such a study should contain a large amount of promoter sequences coupled with high-quality cell-type specific transcript data. If the recent progress made in single cell RNA sequencing in plants could also be adopted to different cassava tissues, this might be an interesting possibility. However, for the time being targeted testing of transgene expression tools will help to identify additional options for cassava.

In this study, we have carefully tested 24 individual promoter sequences for their specificity in stably transformed cassava plants. We find that approximately half of the tested promoters displayed an interesting tissue-specific expression pattern. We especially highlight *pAtCAB1*, *pMePsbR*, *pSlRBCS2* for their activity and specificity in autotrophic tissues, *pAtSUC2*, *pMeSWEET1*, *pMeSUS1* for their activity and specificity in different phloem parts, and *pStPat*, *pStB33*, *pStGBSS1*, and *pMeGPT* for their activity and specificity in heterotrophic storage tissues (starch-storing lower stems and storage roots). Furthermore, *pIbSRD1* represents an interesting option for targeting cambial tissues in cassava.

We hope that these promoter sequences will also facilitate the implementation of cassava biotechnology approaches in other research groups and that these approaches will contribute to positive impact on agriculture in the (sub-)tropics.

## Material and methods

### Plant material and growth conditions

Cassava plants cultivar 60444 were grown from tissue culture in a greenhouse in Erlangen, Germany, or in a confined field at NCHU Taichung, Taiwan. In the greenhouse, a light regime of 12 h light/12 h dark was employed, with a constant temperature of 30°C and 60% relative humidity.

### Cloning

All plasmids were created using Golden Gate cloning. The promoters of *AtCAB1*, *pSlRBCS2*, *AtRBCS3B*, and *pStLS1* were taken from the “MoClo Plant Parts Kit” [Addgene Kit # 1000000047; *pICH45152*, *pICH71301*, *pICH45180*, *pICH41551*; Engler et al. (2014)]. All other promoter elements were created by either PCR amplification or DNA synthesis (All promoter sequences are provided in **Supplementary Table 1** or the supplementary materials). The promoters of *AtCAB1*, *AtGAPA*, *AtFBA2*, *AtRBCS3B*, *MeGBSS1*, *StB33*, *StFBPase_cyt_*, *StLS1*, *StSSS3*, and *StSTP1* were maintained in level 0 promoter modules (GGAT-TACT). The promoters of *AtSUC2*, *CmGolS1*, *CaMV35S*, *CoYMV*, *DjDIO3*, *IbSRD1*, *MeGPT*, *MePsbr*, *AtRBCS1A*, *MeSUS1*, *MeSWEET1-like*, *StGBSS1*, and *StPat* were maintained in level 0 promoter+5´UTR modules (GGAT-AATG). All level 0 promoter modules (GGAT-TACT) were fused with the *Tabacco mosaic virus* 5´UTR [*pICH41402*; Engler et al. (2014)], a modified *beta-glucuronidase* coding sequence [“GUSPlus”; Broothaerts et al. (2005)], the *E. coli* NOPALINE SYNTHASE 3´UTR+terminator [*pICH41421*; Engler et al. (2014)], and the level 1-1f acceptor [*pICH47732*; Engler et al. (2014)] to create the respective promoter-reporter cassette. All level 0 promoter+5´UTR modules (GGAT-AATG) were fused with a modified *beta-glucuronidase* coding sequence [“GUSPlus”; Broothaerts et al. (2005)], the *E. coli* NOPALINE SYNTHASE 3´UTR+terminator [*pICH41421*; Engler et al. (2014)], and the level 1-1f acceptor [*pICH47732*; Engler et al. (2014)] or level 1-3f acceptor [*pICH47751*; Engler et al. (2014)] to create the respective promoter-reporter cassette. The level 1 plasmids containing the respective promoter-reporter cassettes were transferred into the transformation vector *p134GG* (Mehdi et al., 2019) to create the final level 2 transformation plasmids. All promoter-GUS transformation plasmid maps are provided in supplementary material “Plasmid Maps”.

### Cassava transformation

Cassava genotype 60444 was transformed with promoter-reporter constructs as described previously (Bull et al., 2009). Hygromycin-resistant transformants were screened by ß-glucuronidase histological staining (see below). Plants with clear GUS staining were maintained in tissue culture and successively analyzed for their tissue specific expression patterns.

### Histology and microscopy

Different cassava tissues (**Figure S1**) were sampled into ice-cold 90% acetone solution. Leaf-samples were taken with a leaf puncher and cross-sections were manually prepared with a razor blade. These sections were covered with GUS staining buffer (200mM NaP pH7, 100mM K_3_[Fe(CN_6_)], 100mM K_4_[Fe(CN_6_)], 500mM EDTA, 0.5% SILWET^®^ gold) and thoroughly vacuum infiltrated for 10 minutes. The GUS staining buffer was removed and replaced with fresh GUS staining solution containing GUS staining buffer with 0.75mg/ml 5-bromo-4-chloro-3-indolyl-β-D-glucuronic acid (X-Gluc; pre-dissolved in a small amount of DMSO). The GUS staining solution was thoroughly vacuum infiltrated for 10 minutes. The infiltrated tissues were incubated in 37°C overnight or stopped shortly after incubation in case of very quick staining (e.g. *pCoYMV*, *pDjDIO3*). After removal of the GUS staining solution, 70% ethanol was added to the tissue sections and incubated in 37°C until the tissues were cleared. Light microscopic images were taken on a Zeiss Axioskop or a Zeiss STEMI SV11 Stereomicroscope (Zeiss, Wetzlar, Germany).

### Quantification of GUS expression

RNA extraction of cassava source leaves and storage roots was performed using the Spectrum Plant Total RNA Kit (Sigma-Aldrich, St. Louis, MO, USA). cDNA was generated from 0.2-1μg of RNA using RevertAid H Minus Reverse Transcriptase as indicated by the manufacturer (Thermo Fisher Scientific,Waltham, MA, USA). The cDNA was diluted 1:10 and quantification of gene expression was examined using GoTaq^®^ qPCR Master Mix (Promega, Madison, WI, USA). The assay was mixed in a 96-well plate and measured in an AriaMx Real-time PCR System (Agilent, Santa Clara, CA, USA).

The primer pairs “GCGGCCAAAGTCCATCTCCG/TGAAAGCCCGCAACGGTGTC” and “TCTTCGGCGTTAGGAACCCAG/GCAGCCTTATCCTTGTCGGTG” were used to determine *GUS* and *MeGAPDH* expression, respectively. Primer tests were performed and passed (**Figures S5, 6**). The normalized GUS expression of the promoter::GUS lines was determined by the 2^-ΔCt^ calculation method with *MeGAPDH* (*Manes.06g116400*) as a reference gene. The normalized GUS expression of the respective promoter::GUS lines was calculated in relation to the normalized expression of the *pCaMV35S::GUS lines* and displayed as relative expression *pCaMV35S::GUS* lines in percent to provide an approximate classification of expression strength.

## Data availability statement

The original contributions presented in the study are included in the article/**Supplementary Material**. Further inquiries can be directed to the corresponding author.

## Author contributions

WZ performed the experiments and wrote the manuscript. RA transformed all constructs into cassava and provided all transgenic cassava plant lines. CL maintained cassava in tissue culture and assisted the experiments. S-HC managed the cassava field experiment. WG and US supervised the research. All authors contributed to the article and approved the submitted version.
