# Corrigendum: A promoter toolbox for tissue-specific expression supporting translational research in cassava (*Manihot esculenta*)

**DOI:** 10.3389/fpls.2024.1417811

**Published:** 2024-04-30

**Authors:** Wolfgang Zierer, Ravi Bodampalli Anjanappa, Christian Erwin Lamm, Shu-Heng Chang, Wilhelm Gruissem, Uwe Sonnewald

**Affiliations:** ^1^Biochemistry, Department of Biology, Friedrich-Alexander University Erlangen-Nuremberg, Erlangen, Germany; ^2^Plant Biotechnology, Department of Biology, Eidgenössische Technische Hochschule (ETH) Zurich, Zurich, Switzerland; ^3^Advanced Plant Biotechnology Center, National Chung Hsing University, Taichung, Taiwan

**Keywords:** cassava, biotechnology, promoter, storage root, parenchyma, phloem, xylem, tissue

**Error in Figure Legend**

In the published article, there was an error in the legend for Figure 2 and the legend for Figure S2 as published. In the Figure 2 legend, subfigure F_2_ – F_6_ were falsely labeled subfigure H_2_ – H_6_. In the Figure S2 legend, the subfigures C-F were incorrectly labeled and the labeling for subfigures G-H was missing. The corrected legend appears below.

FIGURE 2

Representative GUS staining pattern of at least three events from *pAtCAB1*, *pStLS1*, *pAtRBCS3B*, *pMeGBSS1*, *pStSSS3*, and *pStSTP1* promoter-reporter plants. *pAtCAB1::GUS* = **A_1_**) Source leaf (Inlay = Close-up), **B_1_**) Sink leaf, **C_1_**) Emerging leaves, **D_1_**) Petiole cross-section, **E_1_**) Upper stem cross-section, **F_1_**) Lower stem cross-section, **G_1_**) Storage root cross-section, **H_1_**) Fibrous roots, **I_1_**) GUS expression levels of four *pAtCAB1::GUS* lines relative to three *pCaMV35S::GUS* lines in %. *pStLS1::GUS* = **A_2_**) Source leaf, **B_2_**) Sink leaf, **C_2_**) Petiole cross-section, **D_2_**) Upper stem cross-section, **E_2_**) Storage root cross-section, **F_2_**) Fibrous roots. *pAtRBCS3B::GUS* = **A_3_**) Source leaf, **B_3_**) Sink leaf, **C_3_**) Petiole cross-section, **D_3_**) Upper stem cross-section, **E_3_**) Storage root cross-section, **F_3_**) Fibrous roots. *pMeGBSS1::GUS* = **A_4_**) Source leaf, **B_4_**) Sink leaf, **C_4_**) Petiole cross-section, **D_4_**) Upper stem cross-section, **E_4_**) Storage root cross-section, **F_4_**) Fibrous roots. *pStSSS3::GUS* = **A_5_**) Source leaf, **B_5_**) Sink leaf, **C_5_**) Petiole cross-section, **D_5_**) Upper stem cross-section, **E_5_**) Storage root cross-section, **F_5_**) Fibrous roots. *pStSTP1::GUS* = **A_6_**) Source leaf, **B_6_**) Sink leaf, **C_6_**) Petiole cross-section, **D_6_**) Upper stem cross-section, **E_6_**) Storage root cross-section, **F_6_**) Fibrous roots. Plants were either grown on the field at NCHU experimental station Taichung, Taiwan or in a greenhouse in Erlangen, Germany. Tissues from approximately 3-month-old cassava plants were used.

FIGURE S2

Representative GUS staining pattern of three *pCaMV35S::GUS* promoter-reporter lines. **A**) Source leaf, **B**) Sink leaf, **C**) Emerging leaves, **D**) Petiole cross-section, **E**) Upper stem cross-section, **F**) Lower stem cross-section, **G**) Storage root cross-section, **H**) Fibrous roots.

The authors apologize for these errors and state that this does not change the scientific conclusions of the article in any way. The original article has been updated.

