# Supplementary material for: A promoter toolbox for tissue-specific expression supporting translational research in cassava (Manihot esculenta)
Source: Front Plant Sci. 2022 Dec 20;13:1042379. doi: 10.3389/fpls.2022.1042379 (PMC9807883; doi:10.3389/fpls.2022.1042379)
Supplement: Supplementary file 2 [file DataSheet_2.pdf]

Supplemental Material

Description of sampling position and organ/tissue composition

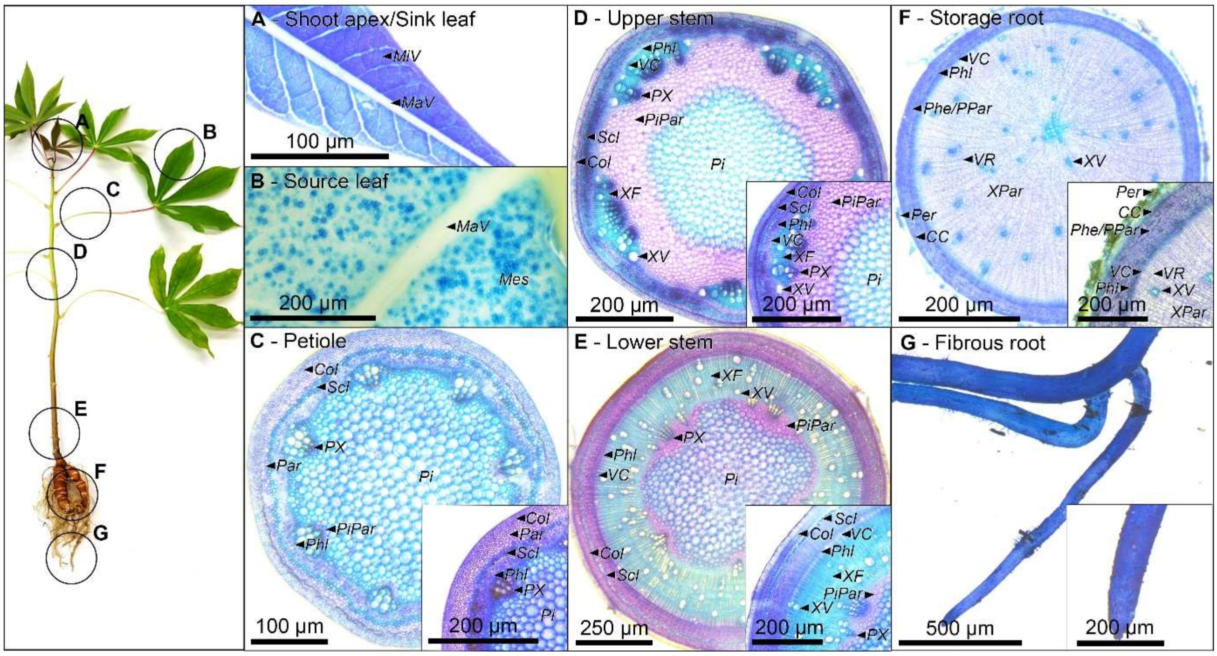

Figure S1_Sampling positions and tissue descriptions. Different organs and organ sections were sampled from three-month-old cassava 60444 plants grown in the greenhouse and counterstained with 10% toluidine blue solution. Numbered circles, in the left figure panel, indicate the approximate sampling position of the respective samples used for GUS staining. The samples were taken from cassava plants transformed with promoter::*uidA* constructs and analyzed for their tissue-specific staining patterns, indicating their respective promoter activity. Note the brownish color of developing (sink) leaves in circle A, easily distinguishable from the green, fully expanded source leaf in circle B. A) Newly emerging leaf/sink leaf, B) Source leaf, C) Petiole cross-section, D) Upper stem cross-section, E) Lower stem cross-section, F) Storage root cross-section, G) Fibrous roots. Abbreviations: Collenchyma (*Col*), Cork cambium (*CC*), Major vein (*MaV*), Mesophyll (*Mes*), Minor vein (*MiV*), Parenchyma (*Par*), Periderm (*Per*), Phelloderm (*Phe*), Phloem Parenchyma (*PPar*), Phloem (*Phl*), Pith Parenchyma (*PiPar*), Pith (*Pi*), Protoxylem (*PX*), Sclerenchyma (*Scl*), Vascular cambium (*VC*), Vascular rays (*VR*), Xylem fiber (*XF*), Xylem parenchyma (*XPar*), Xylem vessel (*XV*).

Characterization of *pCaMV35S* – An ubiquitously active promoter in cassava

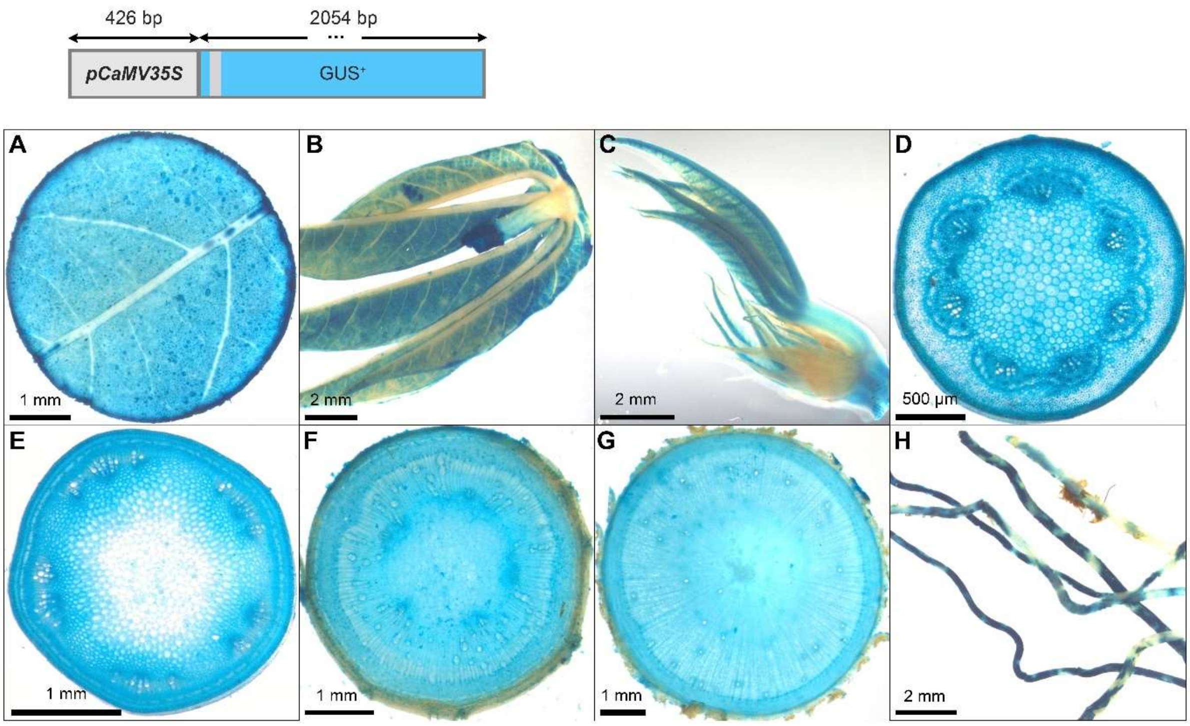

Figure S2_Representative GUS staining pattern of three *pCaMV35S* promoter-reporter lines. A) Source leaf, B) Sink leaf, C) Petiole cross-section, D) Upper stem cross-section, E) Storage root cross-section, F) Fibrous roots.

Characterization of *pStFBPase$_{cyt}$:GUS* – An autotrophic promoter with additional activity in stems and storage roots of cassava

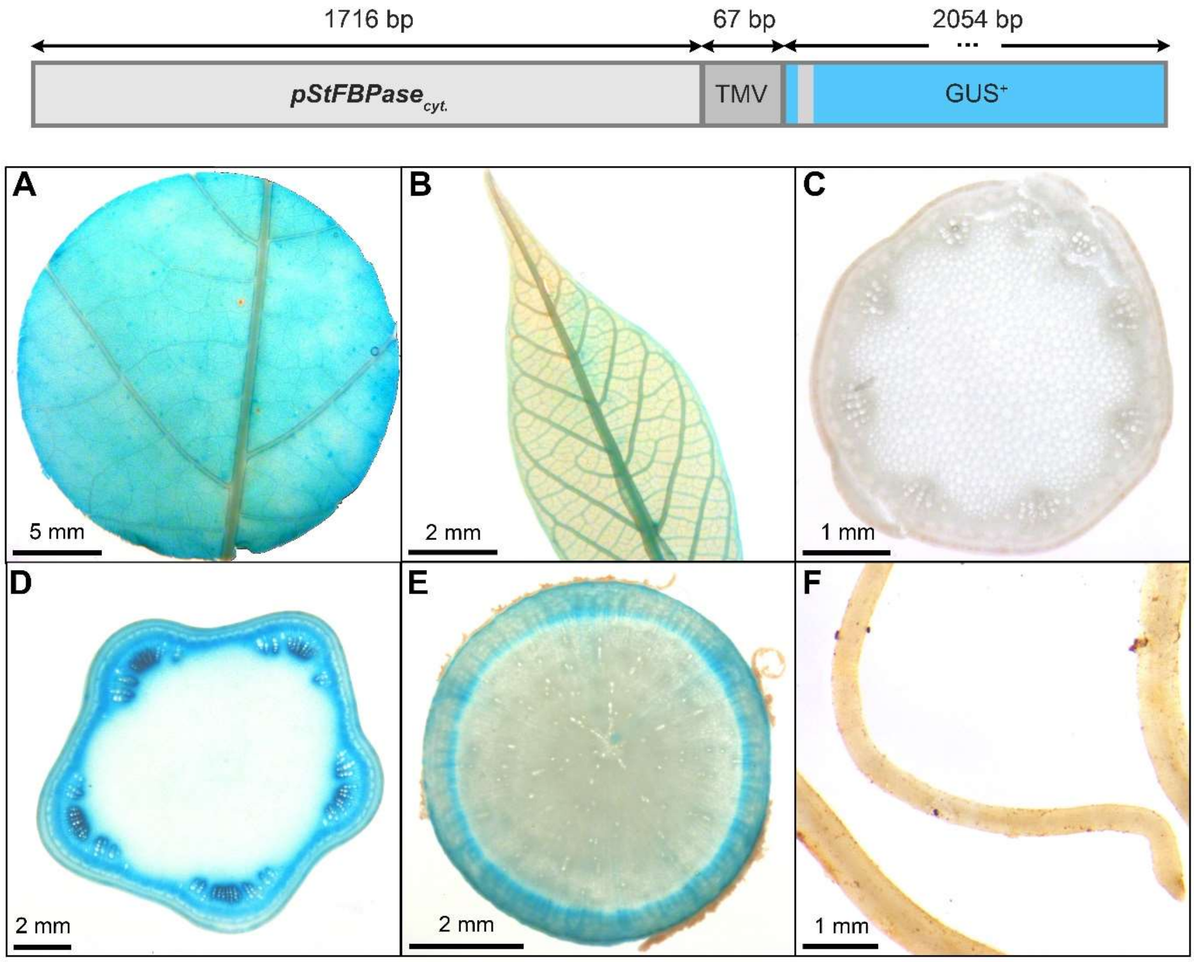

Figure S3_Representative GUS staining pattern of four *pStFBPase$_{cyt}$* promoter-reporter lines. A) Source leaf, B) Sink leaf, C) Petiole cross-section, D) Upper stem cross-section, E) Storage root cross-section, F) Fibrous roots.

Characterization of *pDjDIO3* – A strong but unspecific promoter in cassava

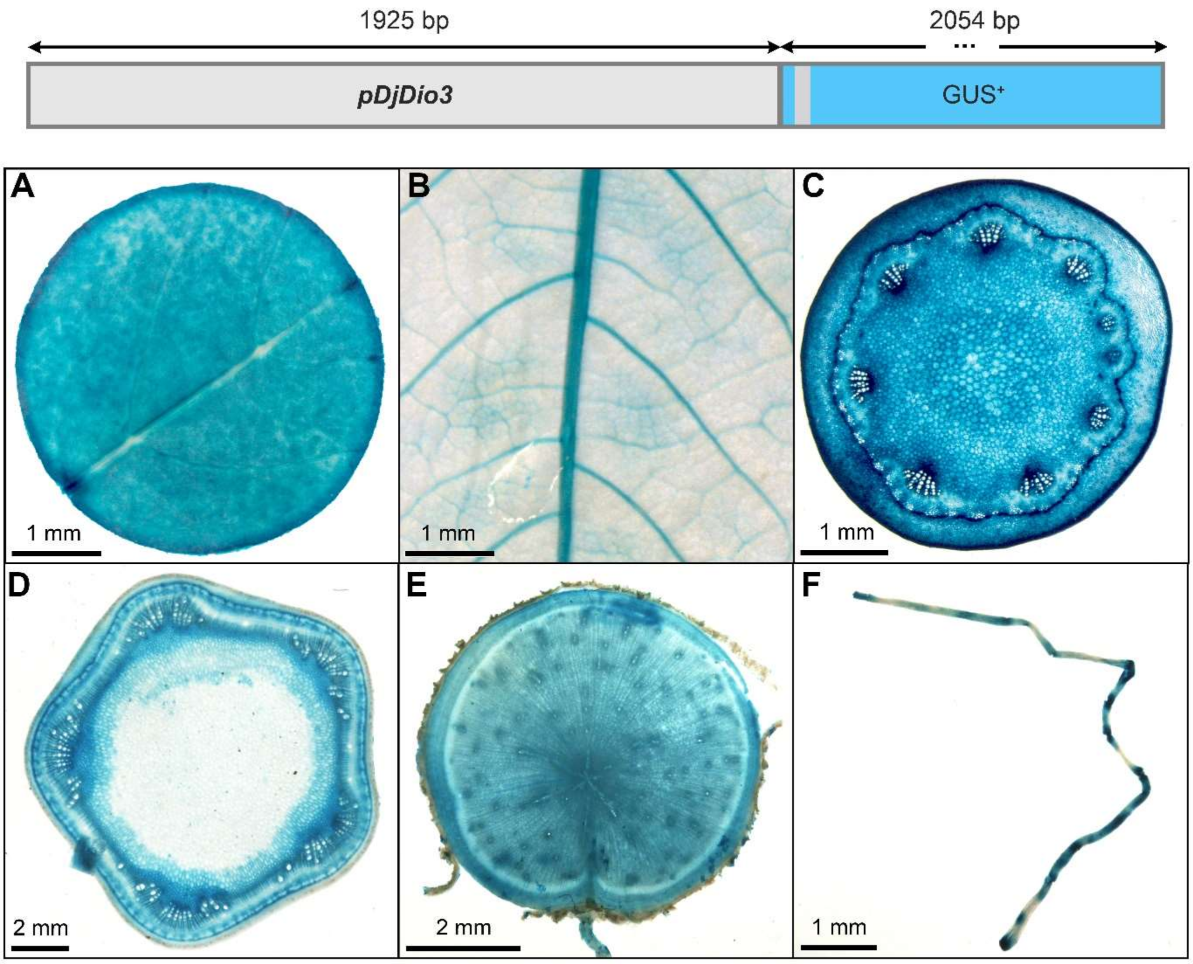

Figure S4_Representative GUS staining pattern of four *pDjDIO3* promoter-reporter lines. A) Source leaf, B) Sink leaf, C) Petiole cross-section, D) Upper stem cross-section, E) Storage root cross-section, F) Fibrous roots.

Primer test "uidA" qRT-PCR

*uidA*_qPCR_fwd	GCGGCCAAAGTCCATCTCCG
*uidA*_qPCR_rev	TGAAAGCCCGCAACGGTGTC

A

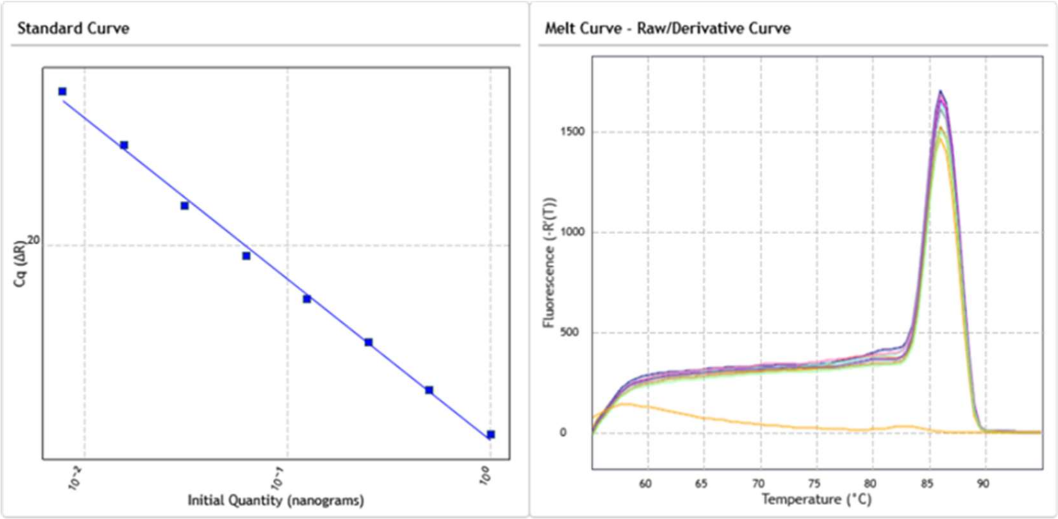

B

Dilution/Control	Dye	Target	Cq (ΔR)	R^2 (ΔR)	Slope (ΔR)	Efficiency (%)	Tm Product 1
"1:1"	SYBR	GUS	15.92	0.997	-3.468	94.2	86
"1:2"	SYBR	GUS	16.89	0.997	-3.468	94.2	86
"1:4"	SYBR	GUS	17.91	0.997	-3.468	94.2	86
"1:8"	SYBR	GUS	18.83	0.997	-3.468	94.2	86
"1:16"	SYBR	GUS	19.76	0.997	-3.468	94.2	86
"1:32"	SYBR	GUS	20.86	0.997	-3.468	94.2	86
"1:64"	SYBR	GUS	22.15	0.997	-3.468	94.2	86
"1:128"	SYBR	GUS	23.29	0.997	-3.468	94.2	86
NTC	SYBR	GUS	38.58	0.997	-3.468	94.2	58

Figure S5_Primer test "*uidA*_qPCR_fwd/rev". **A**) Standard curve and melting curve showing linear performance and desired melting curves. **B**) Tabular results demonstrating a suitable determination coefficient and primer efficiency. Report was created using Agilent "AriaMx" software.

Primer test "*MeGAPDH*" qRT-PCR

*MeGAPDH*_qPCR_fwdTCTTCGGCGTTAGGAACCCAG

*MeGAPDH*_qPCR_rev GCAGCCTTATCCTTGTCGGTG

A

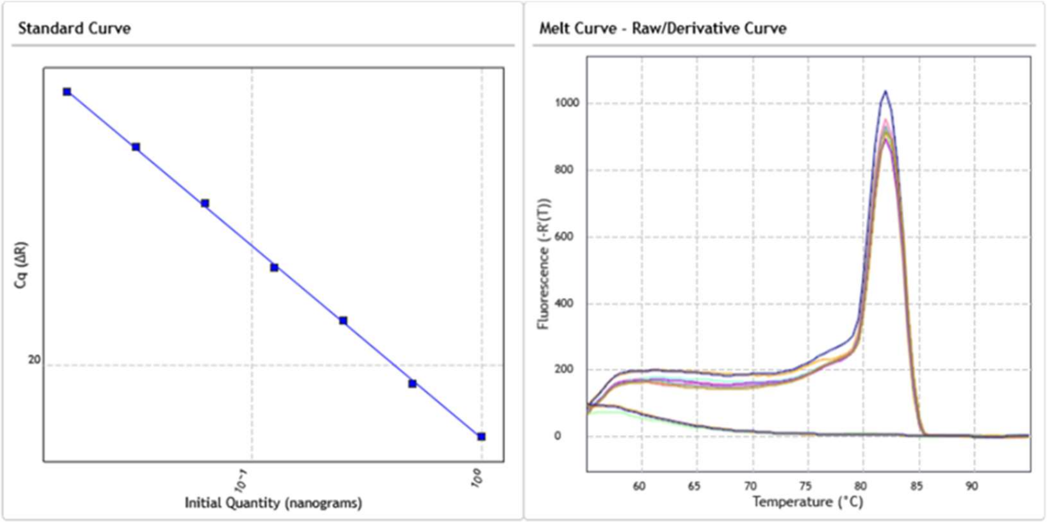

B

Dilution/Control	Dye	Target	Cq (ΔR)	R² (ΔR)	Slope (ΔR)	Efficiency (%)	Tm Product 1
"1:1"	SYBR	GUS	18.74	0.999	-3.379	97.7	82
"1:2"	SYBR	GUS	19.66	0.999	-3.379	97.7	82
"1:4"	SYBR	GUS	20.78	0.999	-3.379	97.7	82
"1:8"	SYBR	GUS	21.7	0.999	-3.379	97.7	82
"1:16"	SYBR	GUS	22.82	0.999	-3.379	97.7	82
"1:32"	SYBR	GUS	23.83	0.999	-3.379	97.7	82
"1:64"	SYBR	GUS	24.78	0.999	-3.379	97.7	82
NTC	SYBR	GUS	No Cq	0.999	-3.379	97.7	57.5

Figure S6_Primer test "*MeGAPDH*_qPCR_fwd/rev". **A**) Standard curve and melting curve showing linear performance and desired melting curves. **B**) Tabular results demonstrating a suitable determination coefficient and primer efficiency. Report was created using Agilent "AriaMx" software.

Promoter sequences used in this study

Solanum tuberosum B33 GENE

```
aaatcattgttttattttctctttctttttacaggtataaaaggtgaaaattgaagcaagattgattg
caagctatgtgtcaccacgttattgatactttggaagaaatttttacttatatgtctttgtttaggag
taatatttgatatgttttagttagattttcttgtcatttatgctttagtataattttagttatttta
ttatatgatcatgggtgaattttgatacaaatattttgtcattaaataaattaatttatcacaactt
gattactttcagtgacaaaaaatgtattgtcgtagtacccttttttgttgaatatgaataattttttt
tattttgtgacaattgtaattgtcactacttatgataatatttagtgacatatatgtcgtcggtaaaa
gcaaacactttcagtgacaaaataatagatttaatcacaaaattattaacctttttttataataataaa
tttatccctaatttatacatttaaggacaaagtattttttttatatataaaaaatagtctttagtgac
gatcgtagtgttgagtctagaaatcataatgttgaatctagaaaaatctcatgcagtgtaaaataaac
ctcaaaaaggacgttcagtccatagaggggtgtatgtgacaccccaacctcagcaaaagaaaacctc
ccttcaacaaggacatttgcggtgctaaacaatttcaagtctcatcacacatatatttattatataat
actaataaagaatagaaaaggaaaggtaaacatcattaaatcgtctttgtatatttttagtgacaact
gattgacgaaatcttttcgtcacacaaaattttagtgacgaaacatgatttatagatgatgaaatt
atttgtccctcataatctaatttgttgtagtgatcattactcctttgtttgttttatttgtcatgtta
gtccattaaaaaaaaatatctctcttcttatgtacgtgaatggttggaacggatctattatataatac
taataaagaatagaaaaaggaaagtgagtgaggttcgagggagagaatctgtttaatatcagagtcga
tcatgtgtcaattttatcgatatgaccctaacttcaactgagtttaaccaattccgataaggcgagaa
atatcatagtattgagtctagaaaaatctcatgtagtgtggggtaaacctcagcaaggacgttgagtc
catagaggggggtgtatgtgacaccccaacctcagcaaaagaaaacctcccctcaagaaggacatttg
cggtgctaaacaatttcaagtctcatcacacatatatatatattatataatactaataaataatagaa
aaaggaaaggtaaacatcactaacgacagttgcggtgcaaactgagtgaggtaataaacatcactaac
ttttattggttatgtcaaactcaaagtaaaatttctcaacttgtttacgtgcctatatataccatgct
tgttatatgctcaaagcaccaacaaaactttcg
```

Manihot esculenta BIDIRECTIONAL SUGAR TRANSPORTER SWEET1

```
ataagacggtatacataacttataaaactaaaatatttataattaggatatatcaatcacatataaat
aattacgaactaattaaatatttttttttcacgtatcgtacgtagaaagtctcacctcaatatgtcac
tatgtgacatgacatattttgctgattatataatttgccttaatactttgaattggggcatataaga
gagtgatttagtttcgtattacataatgactttgattcaaaaattcacaaattattgataatatgaat
atgaaaatcttatatagattaaattaataatatgttataggggatgatatttactaaatcaaaacttt
taaaatattgttgaaattttgattcgaatgtaattaataattcaaaataaaataaatagtatcttatt
attagagttttatttacataaatttattattttatttttaaatattactattaataggttatttcata
tatttttaaaaatttattaaaatattttattttttttattaacaaataatttttttatttaatttc
gtttatttttgtcgttaaatatataataaaaaattaaatttttttaacttttaaaataaaattttaaa
tatataataaaaaattaaaatttttttttatcgattaaaaagtacttttaaaaattaagttaatgaaga
agaagaaagagaaattgagttatccaaaaagaagagaaaaatttgagagtcgcatgcattaaagtcac
agcgtaagtaattaattaatataaatgactatttgagagaaaaaaaaaatagtggttaaaattatttt
tttattaataaattattataaaataaatttacataaattatattaaatttatattttttttattattt
ataagtatttatcgtaataaatatttataacaaaaactttgaatacgaatcattcatatataaagttg
gcattgaattaccatatttgtaaaggaggtaaaaatggtgaggcagttctaagttggatctcaattat
tgcgtgatagttttttttctaattcaacagcacatgagagaggctcatctctgctacctttaacttc
cttatctcatagtttctaattaggacttttcttccctctttttccatttatagtaatttctgattaac
attaatttctatttatcatatttcagaaatttgttttaaaaaatttataaccttgaaaagtttccttt
caaaaagtagatctgaagtgaagtaactttttttataaattaatcaatttttttattaatttactgtt
tactgaaattcaaataggagactttataatcttagggtaagaaattttaactttattaactataaat
gtaaaatttgaaatatgtcattcctttcacaaatattacaattctcacaattattttaaaaataaat
atttaattaataataacatctaaatcaagaatatcaggaaagtttttttttttttttttttaattg
tggtggagttggacttaggtatactagatcttttaattcatctcaacttttgaacaacctcagagaaa
tttacaaataatttcaaaatattataaattagaatatgaatttataatataaatatatagaaattta
```

tataaattttattattattttacagtgtaaaatcacgaatctggctattattttacgctataaatcta
catagaccgcctaatctttttcctcaaggtgcagcgcccattccctgtgagttactgttagcccactg
ttcatagacatcgctcgaattaccaaagcaacttgaaaacaaggaaatttaaacctcgctgttatttg
tttccttctctatataatccattgccacttccctcttccactcatcgcattgacatttgtgaaccaac
ctctgaactttcagtttgattcttcttgaaactatttgtttcttgtctttgccttctcttttcttttg
aaggttatttctttcaatcttgctgaaa

Cauliflower Mosaic Virus 35S

Gtcaacatggtggagcacgacactctggtctactccaaaaatgtcaaagatacagtctcagaagatca
aagggctattgagacttttcaacaaaggataatttcgggaaacctcctcggattccattgcccagcta
tctgtcacttcatcgaaaggacagtagaaaaggaaggtggctcctacaaatgccatcattgcgataaa
ggaaaggctatcattcaagatctctctgccgacagtggtcccaaagatggacccccacccacgaggag
catcgtggaaaaagaagaggttccaaccacgtctacaaagcaagtggattgatgtgacatctccactg
acgtaagggatgacgcacaatcccactatccttcgcaagacccttcctctatataaggaagttcattt
catttggagaggacacgc

Arabidopsis thaliana CHLOROPHYLL A/B-BINDING PROTEIN

Tttccggttgaaatgggtttggtgaatatccggttcagttatatggtggccgttggaattggcttatt
agttgtggccgttgttgaagccgttggtattggtaagggagaagcagacttgtggctatgagtctatg
accatgactcgtgattatggagctgtcttatgaccctgaccatcaccttgatctggtggattccaatg
ttttcttcttcttctaataaaatattatggtcaatacaggtgctaattaagatggtaataatttctta
tgtttctgtggtaaagtttgattcaattccgtagttttagataatcttatttccatacataaattta
tagttttatctactttgttcttatgttttatctctagccaagagttattattattatcagaagaagaa
aaaaaaaagaagcatatatacaaaaggtttaataaaatgtattatacaaggcaattatccaaattttt
tttgttttggtttacattgatgctctcaggatttcataaggatagagagatctattcgtatacgtgtc
acgtcatgagtgggtgtttcgccaatccatgaaacgcacctagatatctaaaacacatatcaattgcg
aatctgcgaagtgcgagccattaaccacgtaagcaaacaaacaatctaaaccccaaaaaaaatctatg
actagccaatagcaacctcagagattgatatttcaagataagacagtatttagatttctgtattatat
atagcgaaaatcgcatcaataccaaaccacc

Comellina Yellow Mottle Virus

Aattcatttcttaggggcttctctcggatgtacaaaaatcaagcttcagccccacatcatttcaaaaa
tatgtgacttctcagatgaaaaactagcaacacctgaaggtatgagaagctggttgggtatcctctca
tatgctagaaattatattcaggatatcggcaaattggtgcaaccactcagacaaaagatggcaccaac
aggagacaagagaatgaatccagaaacatggaagatggtaagacagataaaagaaaaggtgaaaaatc
tccctgatcttcagttaccacctaaagattcattcatcataatagagacggatggttgtatgactggc
tggggagccgtctgcaaatggaaaatgtcaaagcatgatccaagaagcaccgaaagaatttgtgccta
tgctagtggatcattcaatccaataaaatcaaccatcgatgcagagattcaggcggcaatccatggcc
tggataaattcaaaatttattatcttgataaaaaggagctcataattcgctcagactgtgaagcaatt
atcaaattttacaacaagacgaacgaaaataagccgtctagagttagatggttaacattttcagattt
cttaacaggtcttggaatcacagttacattcgagcacatagatggaaagcataatggcttagcagatg
ctctatcaagaatgataaatttcattgtggagaaaaatgatgaatctccatacaggttcacttcatca
gtagaggacgcactaaaggtctgcaatgatgatcacggaagaaatttgatatccgccgtcatcaatga
catcatcacagtactgaggagatgaatacttagccatgaagtagcgtgcgaatattacctatgccttt
attcgcagcgttagtggcactgaaaggcataaagtttgttcgttcttatcaaaaacgaatcttatctt
tgtaacttggttacccggtatgccggttcccaagctttatttccttatttaagcacttgtgtagtagc
ttagaaaaccaacacaacag

Solanum tuberosum CYTOSOLIC FRUCTOSE-1,6-BISPHOSPHATASE

agctaatgctgctcttgtcactcaaaatgatggtatccctctcgtcatccagtgtttgtcaagtcctg
ttaggaacacagtaaggatataaacaaacattttgtggtattcttggttattgagtgcttgctgttca
cttgttaaaattgcacatatacgtagtgagaaactcaactgttgagtaccattgatccgtcaatcttg

tcgataactttgataaggatatttcaggcatcagacatgtcacctctatagaacttggtcttttttttt
taaaaataaaaataaaaatgtttggcatcatacgaacttctgttactttaggctgtatccagaataaa
atgttgtttcctcattctggaattagttgttttgcacacggacgactttcgaaatttactaattgtgt
tcgtccgtctcaaactggctcacactttggtggtcaattttacttctcaaggtaagcaattacagaat
atgaatgtcgctctcctcatatttatccgaacaataaaaaatgatatctgtttgcatatgcatgtaga
tcacacaggggggggggggcggggatctagggagctaaaatctaatttatattagtagatgttgaat
tccgttgggcttcattatgtgtttttacatattcgtttctgaaccacccccaccccggtgaaaaacat
tgctctgccactggctcaatgtattgacacaaatgaacttcaaactgggcaggtgaattatgctctag
gagcattgtattatctatgcaatgcatcaaacaaggaagagatcttaaagccagaagtaattgatgca
atcaaaagttatgcagctgcaggtggagttagtacaagcttcagtaatttggctcaggctttcttaga
tcaacatgttcctcagcttaattaaaatggaggaaaccaaagattatgttgtaaaatcattttctatc
ctagatggtctatcggaaacaatttatttattactcctatccaattcattatattttcaaaagttatg
aagtccacgaaatatgtgacgtgggtaaagacgacccatgccaagccagtgggatatagaaacaaaac
atgtaataaagagaacaaataatgagtttcgaaaagaacagaagttagcataaggacgagaatcacat
tatcttagtgccaaccactaatcctatgtatcattctcctctttccacgtgtcatcctacacttcctt
tgccatcagattagatagcccggttagtacctacactgtatatcaaaaaatacgtaacaatcatccaa
acatatcatcgatcaaaggatatttatcttgatgtgctttcgccgtccattgtaacgagtttggatga
atttgatatacacccactcagatatcaatatattttataaaagaaacaaaattgaatactagtaata
tctatgtagatatttatttttcaacaatcctgtaagttataaggataactcacttatatgtgacgtg
gataatgaagagctaggcaggcagtgagagatagaaacaaattaagcagagacgaaaaacaaatcagt
taacagaatgacgaattggatcacgctttatcttagtgccaaccactgatcccatgcatcactctgct
ctttccacgtggcatcctctgacgtcagatcagattcctcttctttcttttttttttctgtatatata
tgagcattttagtagt

Discorea japonica DISCORIN 3 SMALL SUBUNIT

agacttcgagactccttgatgatatcaaactcctccacttcatgtaaaagttccagcttcttgagttt
aatggagccaaaggaatgtttagcccaatgtcttaaggacccccggagactagcaagtttttcgcaa
gcgtgaacgccccacatcccaccggcgtcggggtaccccaccagagcgcaacgagatcccgaaaccct
tccgcagtgagctaagccaactcgaataggaagggcctaggacgagagaggaaagttcgaagctcgag
gcaaatgggaacatgatcagatcccaagcggggtagactttttggataagcctggggaaaaggtcaa
gccactcactattgacaaggaatctatccaacttaacccagatgggatttgcttggccattggtccac
gtaaactttctacctaccgaaggtggctcccccaaccccaggtccgacacaaaagtggtggcattacg
gaggtcctccaggtttggggtaccggaacacttacaactggggtagaatatcgcattaaaatccccac
aaacaatccacgggacttcaatgccagaaccacaatccctcagctgatcccagaaggcgcacttctga
tcacgagcattaggtccatatacggtagtgcatcgccacaagagattgtctagtaggtcttattttta
cctctttattattttaatttactccttaaaaatacttaagtgaaattaatattcctaaaaatatagat
attgcataaattgctaaagttttagaaggatttatagcatatatactcacgtacacgtatatctggaa
gatttttttaaatttttttatttactttttttatttttttatttttttaaaagagctaccactttagct
ggtttatttttattgagtacaaatcctggacaacatggttacaagaaaacaagcaactaaacatttta
caaatacaaattcaagctaagttgtatggaacgaatggtaaggtcttgtactgagagagatttctgta
gtgttggaatgcgagctctgtctgttgtagggaataataccctctctaacctattggtgtgtgtgttt
tatctactgccttctgtacggctccccatgtatgatatatggcaaccttgctttcagaaaataagaca
aaatattctggtttaaataccacaaaacaaacactaccgtagacatttagaatttctccgctctcaaa
agttaaaagaaaaaaaaagtctattgattctcttctatcatatgtggctgagaggagaaaaacaaagc
cttctaaggcttcatgccttcctaaaatctttgtatcttattttttttatttttgtggattgggatacta
atattaatttgttaaattatgatgacaattgcggaaaaaaaacaatttttgaagattttaaagagtta
tataattttataaataaacctaaaattttcattcaaaaacaggaagtgatatatataaagaccaatat
attgatatacattttgcaattaagttaatacaaattccttcctatttctattgtacaagtgaataaca
agctactcatttccccaaacactgcacacaggaaaaaaaaattattactctcttttgttttcttgttc
tcaggcgaaaaaaaaaaatgaacgaaagaaaatatataccgggtcatatctctctctctcatatatata
tatcttatgaaaagtacttcaagggtgtctatgctattacttgtcttatcaacttctaaatcaacacc
taccacgtctcctagcacttgttatacatggtagagcatctttaattacaaaaccacatccccacaca

ccattaatgaaatctataaattaaagagggcacaactctaaacccactccaatctaagcattcgcaag
gaattaagaaacataacaatc

Arabidopsis thaliana FRUCTOSE-BISPHOSPHATE ALDOLASE 2

Cctacacatccacttggccacctgttgtgcaatgatgacgatgagattgcagtgtctctcgactgatg
atgtagacttcacaatctcttaaaaatcccagtacttaacctcagccttaagaaaacgcagggaacta
cgtcctcacaaaatctttctctttgagtgtaactttcagacgcatctttggctctaaattctaaaaag
gaaaattttaataggttttcataatcatgggttcactggattagcatataagtctatggttgagaaac
ttgagacacagactaacgaaacctctttccggatccaaaggtcctcttgtgtagttgacgtggtaaac
tctctaccgtcaaatttagacattagctaatctgatcaataatctcggcagctcttaaaaattaaaaa
ttagaaatgatacgaacctcataattttctttctcttatcaaaacaccatctttgtatctttataag
cctgttgccactattttaattgaaaatgatgcgtttgtcttatgttttctgtcctggagttcaacat
tatgacaatatgtatagtaaattagtgatatacaagacgtttgcaattcaagaaaaaaacttataaac
taattaatattatggtccacggtgctacatattaactcttgatggttttatacatcttttctacatgc
taatatgcttttaatattgtagcctaacgttataatttgtttttcttaaagaaacagtatcttgaac
gaatcttaactatttctgtacttattcgattttattcgatttttatccgtgtaaaggcaaacgattat
tatgtaacgacgggcataaaaagagtatcgatttcctattcggagaaaaaaaaaaagataaaaattgg
agtgtatgtatatttcttgaattgagagtaatacaagattacggtccaggtggcggaatattattggc
aaggtcacaagaacctcaaataactgatctgaagagaaatataaatcc

Cucumis melo GALACTINOL SYNTHASE 1

tttaatttctaatctaatttgtactagaaaagaaaaaaaaagagtcggattaattctctaccattgag
tggaggatacttggatgcagttcaagttctcatctctccaatttgtcacgtgacagcggatgattaag
catatgagtaggctgcaaaagattatagacgtagaagatgatacccaatacaaaggcgtaacttttcc
cggatgacttttatactctttacaaaattggaagtcctattctatctacatcttaatttccagttgtt
ataatgaagaatagtctgaaaatgatatcaatttttctttctcaataccattcaattacgttaagat
tattaggagctgccattattattattattattgttgttgttattattattattatgcaaccaagtttg
atttgaaattgtttgccaaattttactccaatttgatgttgtttaattactttagatggtataataag
aatgaagttgaatttaaagaaaagaaacaaagcttgaaagaatggaatacttaggtgtagaagatgac
aacgtatttataacgtcgtatagtgtaaataaaaatgcacacatttggatgccctttatgcttcttag
aggtcagactttcccacaaaggctaaggtgattcaatcgtgtgggacatcttgttctcccatttgatt
ctcgttttcattagaccaaaattaacaaaaaaatagtaataattctattctttttaaagtttgtgata
ttacggtttatcctttgttaaaaaagtttatctttgaatgtaagaatttgatagaatgttgaatgaaa
attaagattttgaaaagttttgctgaatttcaaataatataactctctaactttggtttaggaaaatt
aagtgatgacaattatctctattagaattagtattataagtgatatttgagttatgcacttgacttgg
tcgtgttggtaaattctttggatacagaacaaaagaagttgcatgccaagaaagatttctaatagata
tggtgagatatgtggccgttggctctattggattggtggtatgttccagagaagaggagtgcgtatgg
atacgacctaggtggataaatgattatatgaggagatggtaattttatgaaatgtgttagagctttga
tgttaatatatatttttttaagtgtgttttgtgatcgatggtattagatgagttccttattaaacatgt
tttcttggtttttctcgaggtggggttctcaacacttggtaacatgcatcatgtccacgagatgttct
tcatcttatctcttgtaatattatatatgatatctcacacaatacaggttcgtctgaaaaatctttct
ttatttgaaatttttttaggtatttattcttgaggattttttattcttaagtaaagtgttcatgattt
gaagttagaaatataggagttatttttaagagagagtctcacactcaaagggagtctaaatatctttt
ttactaatttaggttgtgtaataaccttgtatttatcgataagtatcacgatgtaatcatttaactat
ctattaacgaaaatctttttttaggacacgttgcctcctagatagatgcaagttgtattgcaaaacttg
tactctgtttttttagtttttttacatgtttactttagaactaaacctaagttatgttatgtgtcaaat
aaacttctttaaaataatattaaaacttctcaaaataataggaaaaaaaaagaaaaatttcaaatttaa
tatatatatatatatattgtaatattagctttcattatcattgaattaaaaattgcatatacaagaat
cgaataatgtggagaaagtagttttcctttttcaactttgtgtagaggctaagtctctaaaatattgg
cttcgactttgtacttttggatccgccaccacaatcagacaaacttccatttgatcattacctttatc
gaatcaaattctttcccttccaatctgtcacaattttgaacataccatccaccttctgatttttttgat
tctaaataaaccttattagcagagattttaaaattagtattaaattataccaaataccctaatgaac

tttttcaatagtttttctattttatttttttttttcttttgtgtgtatgagtttttcaccaccattag
aaaacacatttgaaatatacagaaccaaattgtttaatttgaattggttttccataccattttttacaa
aatacatagtataaccaaaagaactatagttttaagtagtgtataatagtttaattttaaagacaaag
aactaaacaataatcattatcaaaaacactaccttaaaacagaattgaaatcaaatccatttgtttag
gaatatatatatatatatatatatatataatatagtatcataatatataaaaaaaatgtcaaaatctg
agattctttgatcctccctaaattgtccatttttgtcttgcctacaaacttgcaaaaaagaaaaaaaa
aaaggttcatagatagaaatgacccataattgaatcataaagcaataaggatatacaaaattattata
tccaagagggatgagagataatcttaaaggtgcaaaagaatcttcttattgatggaagaagagaatac
aaactcttccaacttttgatcaaaatgcccataatgccctccatctcaccttaaagataggatattcc
aagtcatattcatcccaccaataccaatatctaaaataataagtaacaaataattacaattacaaata
taaagtgcatagaaattaaacttaggggtatctataaacttaaaacaatgttccccaaggctctataa
atagcctccttcccatcccttcacaactcaagcttgaaggactaaaacaagaacttgtaagcttgccc
ttcttattaagtccttcttgcctcccttccttcggagagaaaaaacttttgttgtttcaaaagcacca
aagtcaat

Manihot esculenta GLUCOSE-6-PHOSPHATE/PHOSPHATE TRANSLOCATOR

tgcaattatttaagttggtttctctataataatgaatttaaagattttctaactaaatatttattttt
ctctccctttgaaatattttaattcatattattttattattaaattatttttaataaaatatatta
tttgatatatagcaattaattttaaattttacggcaaatttaaatcgtatataattttttactatccac
attcgatagagattcacgatagttgttcagaaagaaaatttcgagatatataattttttaaaagtgtga
cgagaaccatcagcctaatcacaaaattcgatacaaaaattttatcgtttagaaatttaattttatat
tttaattatttttaaatattttaaatattttcatattttcatatattaaaattttattttctattat
aaatagcatctcttatttattaaaaactcactttgcagaaaaattttaataagagttttaattttta
tcttatttttctcttatttcatcttaacataacaatattgattaaaattctgacggaatttaaatag
ttatttatcattattatttaatttttattttataaaaaaactgactaattaaattatttgatatttta
aaaaatatactatttaattttatttattaaatattataaaaaatttaaaatattttaatatgaaaga
attaattgataaatatcatttcaaaaatatatatattaatttatgattgatagaaaaaaaataattaa
gcatgtatttttgaaatttatacatatattattattattatttaaatcatttatatattttaattaac
aatataagaatcatatagcaaatattctatttaaaaattttaaattattaagattttaattaaattgg
tttttctttaaaaatggtatcaaatcaatattctctttgtatcgaattggaatcaaatcgatttgaat
ttgagaaaaattatttttttttcttttagaaaaattaatcattatattgaatgaaaataaacatttga
aacaaaacactcaaaacagtccattttggggccacatttctttaaaaattttttaagagttaaataagt
tttgatttactaataaaatattatttttataaattaaaatattaaaaaatttagtattttaatcaata
taaaattttttaaaaaatacacagaaatagtaagaaaaataaaatttttattattaaaaaattatattaa
attagaatttatttaaccttgcgcaaatgaggtataattgaaaaagaaaaattaaacttatttaattg
ttagcaaaaattacaaaagcttatttggaatttatttgcaggaaaaatggacggctggaaatacatga
gaaaggggatggaaatatcctcccattcaacaattacatcaatcaatttgtgaaagttggccatgctg
actaaataaaatactattcagaaatttaaaccgtttgaaactttcaaattcttcttgcacggctgtgt
ttagagattcagacattttaaaaatgtggaaatattttatgaagctcggttcaatgaacagaattttg
gaaaaaaaaaaaatttgcctttgatttctcataaaaatccaaataaatgaaatattattacttaaaat
aatattttgttaaattcatttcttattgacttattgtgatgcaggcaatttctaaattgtatcattgc
ttcacagactgagagtcatggcctcttcccatataaacaccctctcctccaacctctctccagtagca
tacagcttcctttcatctccaatcccgacaacccctctcctgtcctcacttcttcacaattctggttt
tttctcctatatatatttcccctacattctcatattttcactggaaacaaacagagtttcaagaaatt
ctctttcaagtattctttatctgtgtgcttcttttgataccagtaacgttaacacattcttttgtttg
tagctgtaagcctgtactctactagcac

Arabidopsis thaliana GLYCERALDEHYDE-3-PHOSPHATE DEHYDROGENASE SUBUNIT A

Cctgcaggcatcaggctagtgtcaatgctatagcttgggctcctcatagctcttcccatatctgctcc
gctggagatgattctcaggcgttgatttgggatatatcttccatgggacagcatgttgaaggtggtct
ggatccgattctagcttacacagccggcgctgaggttgagcagcttcagtggtcatcttctcagcctg
attgggttgccattgccttctctaacaagctgcagattctccgggtctgattatgttttacagatttc

tgttggatacttggatgtaaaaactcgttgctattgttgattaatgatcttttgatctctgactctaa
gcctcattgagtttcctttcatagtatggtctgtgtaggaactatgtaccttcaaaattgtgaataac
tagctttagcattggatttgattagacgtatggaagttagttttgaacagagagcatgtgacataacg
gggaacttgaagtagattatgactaacaccagcatttatgtttctctgccctcgagctgatggagttg
caggattttgtctgcctcaccatcgtatggaatgtgtagctttctatggtagttgttataagttttgt
ctccattgtccatatcactttatgggtatgtagatttgtgaaattaggcatttgtaacatcgtacaga
tgatgtattcttcattacttcaatacaggcatatagactctctgtccaacttaaaggcatacaaggt
ctgtggtttaccaaaatcagctttactaaatcctttggccaatattctctcagccagtcctaagaacc
tctgctctaaaaagtcttagctttgaaagtcagacaaatggagagcagccttatcctccttttataac
cgtgaccacacacacaaatgaagaagcccaaatgaaaggcccaaaaatctaaaaaataggtgaaggag
ggactttgtgaatatgtgatcatagaaacatgaagagacaagagagtggtaaaacc

Solanum tuberosum GRANULE-BOUND STARCH SYNTHASE1

aagctttaacgagatagaaaattataatactccgttttgttcattacttaacaaatgcaacagtatct
tgtaccaaatcctttctctcttttcaaacttttctatttggctgttgacagagtaatcaggatacaaa
ccacaagtatttaattgactcatccaccagatattatgatttatgaatcctcgaaaagcctatccatt
aagtcctcatctatggatatacttgacagtttcttcctatttgggtatttttttcctgccaagtggaa
cggagacatgttatgttgtatacgggaagctcgttaaaaaaaaaatacaataggaagaaatgtaacaa
acattgaatgttgtttttaaccatccttccttttagcagtgtaccaattttgtaatagaaccatgcat
ctcaatcttaatactaaaaaatgcaacaaaattctagtggagggaccagtaccagtacattagatatt
atttttattactataataataatttaactaacacgagacataggaatgtcaagtggtagcggtagga
gggagttggtttagtttttagatactaggagacagaaccggagggggcccattgcaaggcccaagttg
aagtccagccgtgaatcaacaaagagagggcccataatactgttgatgagcatttccctataatacag
tgtccacagttgccttccgctaagggatagccacccgctattctcttgacacgtgtcactgaaacctg
ctacaaataaggcaggcacctcctcattctcacactcactcactcactcactcactcacacagctcaa
caagtggtaacttttactcatctcctccaattatttctgatttcatgcatgtttccctacattctatt
atgaatcgtgttatggtgtataaacgttgtttcatatctcatctcatctattctgattttgattctct
tgcctactgtaatcggtgataaatgtgaatgcttcctcttcttcttcttcttctcagaaatcaatttc
tgttttgtttttgttcatctgtagcttggtagattccccttttttgtagaccacacatcac

Manihot esculenta GRANULE-BOUND STARCH SYNTHASE1

gtggaatggagattagtagcatcatagcgtgaactctcggcttttagatttagggcaaatataagggt
acacttcaaaacgacgtagtataggtgatttcggttcggttcggtttaatcgaaattttgaatctaa
aattgaaccgaaccaaaatcaccgatttttcataaaattaaattcaaatcgaatcgaattaaaaataa
aaccgaaccaaatttttgattcggttcagttcgatcggttatttcggtttaaaccgaatagtgctcac
ccctagcgagtgtaactttaatctattttaaaattaaaattaaaattatttattttattataataat
tataaatatttataataaatatatcattaatttaaaatagcagtaggtataaaataattttaaaaaa
atttatttattataaattcttcttaattattactttaaaattacattatttactattgaaatcgtaaa
ttatacatttaagaattagattatcattgtctttaatattaaataaataaattaattataattaaata
tgcattaatattttcatttataaaattaatttatatttaaaaataataaaatattatatttaacttgg
ttcaaaaaaatattatatttaatatattatttaaaatttaaaattttaattttaaaaattaattaaaa
ttatatttaaatataaatattaaatgtttaaattaaaatgtgaaataattaaaatgtggactttttt
tccttataaaaatcatatatgcacggaacaaacacaaccgtccattgttcaatcaatgaattgaaatg
taggaccctggcgaagtcccaccattactaatctcaaccacaacacaagtagaggtagaggtgttgag
ctagtaaacttatagcatattcccactgatacccctttaacctatgctaatgcaaattgccacaatggg
tcccacttcacgtgctctctcccttgggaggtgcccagtatgttgggagtattttctcaaccagtacc
gaagcgacacgtgtacatcaccaacagctacaatactcaccttcctccctagcaaaatctttcagtgg
atcatcatattatagcatatcaaatctccactccaccacaaccaccagcggaacctattttgcgccta
agcttgt

Solanum tuberosum LEAF-SPECIFIC1

gtgttcatctgttggacaagctaataatatctatttgttgtctgaatcacaattttcccatgataaat
taaagccaaaattcggcttctcacaacctgatagaatctaattttatgacttttatcaaaaaaaagaa
gaagatagaatctaattttatgacatttaatggtagccattccttaaaaattcccgattcaactaatc
cttggagttcattttctcataggtggatttcatgctggagttactgtaaatagggttggatttctaca
ttcttgcgagagtgatctgcattccagcccctctgaagcacaggagtcagctgcatttcaaatgatt
acccaattacagactatggcagaccgcacccattagatttaatgtggcaaagaaacatcaactccaat
atttttgaatcctattaatggctatcataccatagtactgtattagtactgtccagaagcaaaaattg
ttgttttagaatattacttggttgcaaccgtatcagtgctttgaacatttatcaaggccaaatacata
atggtgccttgaaatcaactctagtttattgaaatgatgtctattgagcacctatatgtgttgccagt
attgcccatgcttaacttagcattctgttaacaagtgttatgctggatgttcgaatcgaacatggagt
tttcatgttgcttagaactatatgctttcagtgttagcagtatcggaaggtaaatacagtttaaacac
caaaagtagagagaaataaggaggaatttaatctttggatttgattcgtctttcttgtgggaatggtg
aactcgttaaccagctaggtgaaatgaactcaatacttagttcaaaattgtttaagttccatttttgg
ttaagagaattgagttgtgctctctagcttttgaagatgatttaggttcaaggactataattttacac
gatattagagctagatccattgataggccgcgtttcttaatgtttttggccttgtaattctgttttca
cattgattgactgaatgagttgtgctctttctatatgatctcggataattctttttctcatgagctaa
cttaagttaggatatgagctaactgctctccaacaggcaacaacccatagaggaaccaatcagttttt
tctttttgtcctcctcttccttgcacgagtggacttagtaaatagaaaggaaaataacaaaaatgtat
aagaaacaagtcaatctcttgggtcccttaataatcaaagaaaaccaatgaatgaactccaagtgcac
aaaactaagataaggcaaaggaaaataatttgtagtaagaatgctatatattgataatgttggtaata
atattatctagaagcaatccacagaaggggtgccacgtgtcaacttgtaaatcacaaattgcaagtca
tcactaatattattatcaagggaccatgcaaagtgaaaataaataattcatactaagtagtgagagca
atact

Ipomoea batatas MADS-BOX PROTEIN SRD1

ggctggtttctaagacatttttggtttaatccaaacctaattacaaatattcccaacaagatcgaat
gatctatggctacaaaccctatcccaacaaaaaactacatttagtacatcaaattaagtggcatgatt
attttattttgttcgacaaagtagcatcaaataaactacaaaaaaaactacatcattacaaaaagact
aattatcaggcatcaatgttagtatatgggaggtggtgggttcgagcctcagtggaggcgttgctgtc
tctttgttcttcagtaggttgagagagtaatttatgaacagatactacactgtaatagagtcagtagc
aatcaaaaaaaaatttgttttaataatatcctaatattatatttttctaaccagtactatgctttcgg
ctttccagaaggcagaagcctaaaaaattcaattaagtttataaactttaatccacttgtttgagtaa
ttgagtatctttcagaacggttgtagatttaggtgggatgacaaatggtattccaaagttcaagatat
ttctttttagatttaggaatttgtagtcttttaaggttagaggttacttaaaaggatgaacaaatttt
ttatcccattctatttctaggaagaatttataatccgtacgtgtgacggctgccattaattatagtgc
ccattcattttattgggaaaaagtactcatccattatttcattggcacggcaacccagttttaaata
ttttataacaataataacatatggaaccaaattgtaacctttatatcccacagacccacacattacac
atccaataaaacttgagccaaattatatattagcgttactgagtactgactaaaatatatttttaaaa
tatactaaaatattattaaaaaaatattaaaatagtaaaattatattaaatagaaaatttaatttaat
caaagaataccaactaaaacgtataaaatgagaaaatataggtatataatattgatgattcctttga
tttttttttatgatccgaaaattctttggccataagaagagtaaatgaacaatttaaactaagaaaa
taagtaagttgctcctatgtgattaatatataaagtgagatttgagctgttgatctatattattgaat
tagatcaacgactcaaaatgaaggataatttttttaaaaaatcgcttcctgttaatattaatgcttta
aaattaagcacattaaactttaaaataatgcaccttttttttttaatactattgaccttgttacatgta
gtatctgaagtccaacaaagtcaacattgtccccactgaggctcaaacccgtgacctcccactaggga
gaatcgcttcatgccgcttgaccacaagtcctttggtaaaaataatgcaccttaaagatgtaaactta
cgcatcttcgatgaactgaccactttgagcttgcaacttatactttttttgaagataagcttgtaactt
attataatggtctattaacattaaaaaaaaaaaaagtttcacaatcaaattataatatttgtagccaa
atgaatttaccgcgggtgtgactattcaggaatttaaatacactaaagttggagggtagtacactca
atacactattgctcatgactttttcttcttttttttttagattagctaatatattaatcccaaatag
aaacgtttacaccaaagttcgaaaaaatgttgtgtcatttcttacagttagacacaaaaataacattt
ttagctaagttacagtaaacttgattggcagactgtttcacaaattgggagcttggatccttgaagga

acttactgctttcttagagtcattaatggtttggccaaacatagaaaagattagttgagcagtcttgc
acactacttgagtaatcatctccattcttctacttattgacaatattctcttatgaaaaacacactt
gatcttatatcagttagggatttgaccggtttattaaaggatagcctaccaactttgttgaacgacat
atcatcatatcatgattcaaaagatgctctttttattgtcatatttgtggcacaggatgagtacagt
ttcgcatacaccatgatcattttatcaaatcatactctataaaccctgtcaaagaaaagagaggaa
gaaacgagaagaagaaactcatccaagaaacaagaggaacattattgctcatgattagatcgacttga
acatgtactaatgccaatctcaaattacctacataggtgtgttagacaaatatttgttaattagctga
ttgacttaatggatttgactagttgttaacattaattgattgtaggaaattgtttggtaaattagttg
ttagttgatagttgattacatgaaaattactttctcaaaaagcttatcgaaaaaactattttgaacag
ctttttgaattttaacattttataacaataagttgttacaaaaagctaattaatcaaatactcatatc
cattgtttaaccatgtcaaacaactaataataattaaataatttgtttttaaaatataagttaaattt
aattgataagctaactatattaccaaacataccgtaatattttcttaaccgcggtatggctaagata
tgattgtatactatttttgttgcgagcatgattaatacagtaataccatcatttaaaagtggaaacca
cattcgcagctgtttccgaaagcaaacagctaacatttgctaggttcttacttatgcattaatctggg
ttataaaatccccatttccatgttggtgtgaacaaccacctaaacctagcgtgttcaacaattctacc
ctactatcatcccccaagacttccccgaccagtaaataacccgctttcctctttcagtgatttcttca
tttgactttgctatatatatatataatctgatctgctttcatctttcagtgatttcttcatttggatt
tcttcagaggaggagaagg

Solanum tuberosum PATATIN CLASS I

tgcgtattagttttagcgacgaagcactaaatcgtctttgtatactttgagtgacacatgtttagtga
cgactgattgacgaaatttttttcgtctcacaaaattttagtgacgaaacatgatttatagatgacg
aaattatttgtccctcataatctaatttgttgtagtgatcattactcctttgtttgttttatttgtca
tgttagttcattaaaaaaaaaatctctcttcttatcaatcctgacgtgtttaatatcataagattaaa
aaatattttaatatatctttaatttaaactcacaaagtttaatttcttcgttaacttaatttgtcaa
atcaggctcaaagatcgtttttcatatcggaatgaggattttatttattcttttaaaaataaagaggt
gttgagctaaacaatttcaaatctcatcacacatatggggtcagccacaaaaataaagaacggttgga
acggatctattatataatactaataaagaatagaaaaaggaaagtgagtgaggtgcgagggagagaat
ctgtttaatatcagagtcgatcatgtgtcagttttatcgatatgactttgacttcaactgagtttaag
caattctgataaggcgaggaaaatcacagtgctgaatctagaaaaatcttatacaatgtgagataaat
ctcaacaaaaacgttgagtccatagaggggtgtatgtgacaccccaacctcagcaaaagaaaacctc
ccctcaagaaggacatttgcggtgctaaacaatttcaagtctcatcacacatatatattatataatac
taataaagaatagaaaaaggaaaggtaaacatcactaatgacagttgcggtgcaaagtgagtgagata
ataaacatcagtaatagacatcactaacttttattggttatgtcaaactcaaaataaaatttctcaac
ttgtttacgtgcctatatataccatgcttgttatatgccatggtacc

Manihot esculenta PHOTOSYSTEM II SUBUNIT R

catgtgaatggtgggtaaaataggcaaagtaaaaaaaccaaaaaaaaagtcaactagcaaaggcgtcgt
catgactgattcagctgcgttttttttttttttttttgggaatttgtagacttaggaggaaggaat
tcagcaaggacgaggaagcgagaatctatctatgctcgttccttcctttatttttttttaattaattt
ttttaggcacaattctcacatgttgtaaatataatattttaaattacttaattactttcttataatt
tttaattaccggaaagatcaaaataattcaacctcaattttcaatgtttaatatgttatactcaaagt
ttgacggctgaatatattataaaaattgatgtgcgaggagattttttaagggtttttaaatttttttcat
attatatacgaattttttttttattaaagcaaatgatatttatttataaatatacagaggcgccatgat
aacagtgagataagttagatttagcaagatattcaaataagaatatttagataaagatttaatttatc
ggattaattatcgttctgtaataagttcaatatttgactttaaaaaataaaatctatttaatatttga
ctttaaaaaataaaatttattaaataaaaaagacggttaagagatcactaaaaaattttatgtgaaaa
ctctccgatactcaaattagaattgaaaacttcacgaaattaaggaaagtaaagcagagagagaaaat
aagagaatggtggttttgaattcttcatgagtgaccgttcgagagaaatgtgtctttttttcagagaat
gagagatctcttttttagaattagtttgatacataaatacttttttaataaaattgatgtccctagtgt
cacatttccttctaatagaatagagatattttttgtgtagatcaggtattacattaatgatggagtata
ggataataaatgtgaaactgatcggttcatacctttttatattaaatgggcgtttatgatttttttggac

taaattaaagaggcgataaatttatagaagattgttatttttaccctgacttcatactgactcatccg
acctatatagaaataaaccttttttcagttaatgaaatcagccacatcagcgttttaaactttgttgta
gtaaagattttaaggaggtcgatctgtttgtcatttctagtctttgagaaggtaaatctatatgttc
cggacttaaataaatttaagagtgttgctcgtatgatttttatactcaatagattgaatttcttatcg
aaaagattttcaattcacatatacaaatataatatatatatatagtattttatttttttatacattatc
atgctgtaattaaattaaaaaaatatatattgtcacaatttgaattttaacacttgtttaaaattgtg
ccatctttaatttgaatttattttcgatcaaatcaatcgatatattcacatattcatctttaatttaa
atttagttttatcattatgagattcaaattatattataattaaaaaaataaagaaaatatctaataaa
ccttattattttgattagtttttggatttattagttttattatataaaaaagtgattaatagattcaa
ttaatttgtaataatttattgtatagtataaaattgataaattaattgaataaagcagaatggaatac
gtaaatactgtcttatgctttattaaacataaattttataattttttttgagagaataaattttataa
ttaatgatgtagaagattattgcaaatgtttaaatgtttaccactaaataaaataaattttttttata
aataattagaaccaatggagtgactccacgcgtgcaaggcatggatgaaaggtgaaggatttgcaacc
acccatcctgataacgtttgcaacatcatatccagatataaaaccacaaaacctggacacgtggtgaa
gtgagaatcactattcagtaatttaattatttatttatttatttatcagtgcaacattttacttgaa
aagattgaaaggcaaagaagagagaagagaagaaaacaa

Arabidopsis thaliana RIBULOSE BISPHOSPHATE CARBOXYLASE SMALL SUBUNIT 1A

aacattcatacgttttcatttgaaatatcctctataattatattgaatttggcacataataagaaacc
taattggtgatttattttactagtaaatttctggtgatgggctttctactagaaagctctcggaaaat
cttggaccaaatccatattccatgacttcgattgttaaccctattagttttcacaaacatactatcaa
tatcattgcaacggaaaaggtacaagtaaaacattcaatccgatagggaagtgatgtaggaggttggc
aagacaggcccagaaagagatttatctgacttgttttgtgtatagttttcaatgttcataaaggaaga
tggagacttgagaagtttttttttggactttgtttagctttgttgggcgtttttttttttttgatcaata
actttgttgggcttatgatttgtaatattttcgtggactctttagtttatttagacgtgctaactttg
ttgggcttatgacttgttgtaacatattgtaacagatgacttgatgtgcgactaatctttacacatta
aacatagttctgtttttttgaaagttcttattttcatttttatttgaatgttatatatttttctatatt
tataattctagtaaaaggcaaattttgcttttaaatgaaaaaaatatatattccacagtttcacctaa
tcttatgcatttagcagtacaaattcaaaaatttcccatttttattcatgaatcataccattatatat
taactaaatccaaggtaaaaaaaaggtatgaaagctctatagtaagtaaaatataaattccccataag
gaaagggccaagtccaccaggcaagtaaaatgagcaagcaccactccaccatcacacaatttcactca
tagataacgataagattcatggaattatcttccacgtggcattattccagcggttcaagccgataagc
gtctcaacacctctccttaggcctttgtggccgttaccaagtaaaattaacctcacacatatccacac
tcaaaatccaacggtgtagatcctagtccacttgaatctcatgtatcctagaccctccgatcactcca
aagcttgttctcattgttgttatcattatatatagatgaccaaagcactagaccaaacctcagtcaca
caaagagtaaagaagaaca

Solanum lycopersicum RIBULOSE BISPHOSPHATE CARBOXYLASE SMALL SUBUNIT 2

Aattcatggggcattacaattaattagtaatactaattttaataacactaaatcatattagtctctat
aggtataatatataactctggagtcactatctgtatttgggatcaacagttacaaaggtacgagtaaa
gtctctgatccgacaaacaatccgtatatcctaccttcccgaaatttcatgtataaaattatactaaa
atattatttttcttgtattatccgattgaaaattatgcgaattcaacataaaactctaaaaaaagaaa
ataatgaaaattacaacttaaaagagtgtctaagttggaatcatggtattacataaagttgaggacaa
tataagcaagcaaacaagtactctagctatcaatttactttggactactatatgataaatatttcaac
ccccctcctccccccaccaaaaaaaaattaaaataacaagttgaaggactcaaaaaaaaaaaaaaaaaa
aactcaaaaccaacctcaatcatacattcatatcctcttcctacccccatcttggatgagataagatt
aacgaggtgcttacacgtgtcacctctattgtggtgacttaaaaaaaattccaacctttcatatgtag
atattaagtaattgtataatgttatcaagaaccacataacatatcaaaaaccttatcatttcattata
taaaaggatagtggacatcaaaaggttcatattgaaccaaaaaaagagagaagaagcaa

Arabidopsis thaliana RIBULOSE BISPHOSPHATE CARBOXYLASE SMALL SUBUNIT 3

Gttagaattcttttgataggtttacttccattagtctgaaatggaatcttatttataagtctgcctga
aaaatcttaacaattgatattaataaaacggaaagagtaatgaatatttttaagagcatctcgaatct
atctctatttttatttatatactctatttttgtatttatttaactctaactcatctctattttcattt
gtaaaatagaaatttctaatttttttctaaagcaaaaaatagagattttttatttttctcttattta
tttttctaacgaattttgaggaaccattgaagttaaaatataaatctctattatatttttcctct
atattcagaaagaaaataatagaaacaaatattggagatggttaaagcttttttttttgtccggataa
atatcatacaatatatttatgtaaatatcaaatttaaataattttgttataactatttttaatgcga
ccttatttccaaagaaagtgttaagccaaattaaatcatacatggtaaataagaggtccaaaagacca
aaagcattaaatttaaggtaatgcgtctggtttcttttctcccaaacgaaaggagccaaaagcaaccg
atcaagtgcagaccagtaaccgcacattcacttcatttcccccacaagagaaaagataagataatgga
gtttctgccacgtggccttatcctagtggtgcgtaccgataagggtgtcaacacctttccttaatcc
tgtggcagtaaacgacgttatcatgaatcatggaccctttgatcatgagggc

Solanum tuberosum SOLUBE STARCH SYNTHASE 3

Ggaagaaatcttctctgtctaaaaaattgacggttgatgttataagagttttttttttttttttttt
ccttttacaaattgaagtgacaaaagagtgaatcacagtggatcgtgataacaaagaatgttctgaga
gtattgctattgtgaattttatttatatgacgtaaatggtcaaatttgcttaattttgtgaaataga
acaaatttatcctctagttggagacaaatatactcttgaactatacaaaatagaataaatttttgtt
atttttaaatttatcgcccaatgatattaaagatagtttttttttttttaaatgaattgaaactttc
gcatagttcaattttttttcttgcacaatatataggaaaatttaatccttctttctagttttgaatt
ttagaaatcttttcttcctcctttctctctctaccacccattaccccttttttctccttcttccttg
aacaacgccatacaatttttatttctttttcccctcatttttttttaattccaatcaaattgtat
aatccaagtatagttaattttgataacatataaattataaagtaatgtgtaaataatagtactatagt
ataataagttaaataaatcaaactcaaataaatttagaccgctgctgtcactctctaggctctccata
gccaaattttgtcgcaaccttttttgctcatttccaagaaaaaaaagaagcctactgtaatttaaaaa
attccacgtgtcaatatctaaattaccaccgtcatatcgaaccaaacaatttcaaccgtcagatttac
aggtacaacgtaagcaaaatagagagcattacacagaaacaacacacttcaggctgaaatttgtagga
ctcctccaatttgttatcgcagttcgtagaatctgaagaaagagtattattcttgatttttttaataga
tttttaaaaccccattaaagcaaatacgtatataattgcagcacagatacagagagggagaga

Solanum tuberosum STARCH PHOSPHORYLASE 1

tcaagagtcaatgatcccaatattttaacggatactattagctttactttattatttttgactttc
ctgttaagtataagatttgaaatgaatctcaatcatagtttattttattatccatatgacgttgattt
aagagtcattgaacattacatgaggaaatagtacttgactgggatcttctttcatctaaactgacact
aaactctttttcttcccttctccaatatccaacatgcaattagacgatgaacgaaatgtgatgaaaa
atttgataaatgagagttcaaattttaacaaaattaaataaaaaacataatcaatttttaaatttta
gaaatagagttattgtttaaatgatacattgaaattgcagtatatatcttatgaaataatggagataa
cttaaattgaccaaacattattattatttacacaaaaggggggaaatagcaattttggaccaaatatt
atactaaggaataggatgaaattataaaatgatttgctcgtttttttttcttctcaaaaacgaaagaa
ccgcacaagttgcggatctcatgagatcattacccaatgcattaggtagagtaagatccacatcacta
acctttctccgtcaattttatttggcccatatattaaaaaaatatttatttaaaaaattagaagct
aatatattattatgaagtttaatttattgttattattaactatagtaattatttcaagtatattttt
aaaatattaaatttattatattcgaaagaagatgtaataaatgtatcaatctttctgtttcaatttat
ataattcatgttattttagtttgcctaaaaagaatgatacatttgcagtggtgacacgatttgtaaaa
atttatgcgtactcattgtctatatgtatgtatcgcagcggcaagcgagatgaaagagatgcaagaag
atttgttatctatttcaaaatatatatgaatcttacttagacacaatgtatatagaacaaattatatg
taatagttgaccctatatatgtggtaaaatacttgactattaggggttgtttggtagagtgtattaag
aaatataatgcatatattaggtgtgtgtattagtagtaccttgtttggcacactttttcatgccatgt
ataactaatgcatgtgtattactaataccaaggaattctaggtattagtaataaatagcattttaaca
cttgcattagatcaaataattacaaaactacccttaaagcattttcattttctttgttgtcataagtt
tttattttttattttatttgcttttcggtatcttttaatttgttggtgtcttaatagactttatggcc
ttttaagtatctttttaaaaaaaatctaatttcaatataatttaaattttttttttactattgtgacaa

taaatttgataaaaaaattatttgccaactttcacaaaaatattttgacgcaatagtataactattt
aatactatttttttatttttttatttataaaaaagatgaagagttaatgatgttttaacaaagatttttt
tttttgatgttttagcaaaaaactttcttgcaaaggaagtgtacaaataaataaagtgtgaagggtat
ttttgtaaacatatattatttaatagtaattatgcaagatttattattttttaatacatcaaaccaaac
aatgtataagaaataatacttgcataactaatgcacgcactactaatgcaagcattactaatgcacca
tattttgtatttgttcttatacactctaccaaacgaccccttagagtgtgggtaagtaattaagttag
ggatttgtgggaaatggacaaatataagagagtgcaggggagtagtgcaggagattttcgtgcttta
ttgataaataaaaaaagggtgacatttaatttccacaagaggacgcaacacaacacacttaattcctg
tgtgtgaatcaataattgacttctccaatcttcatcaataaaataattcacaatcctcactctcttat
cactctcattcgaaaagctagatttgcatagagagcacaaa

Manihot esculenta SUCROSE SYNTHASE 1

tggcattgcatcagttgttttactctttcaggatattcctctacgattcttgtttcaacagagttgga
tgaccaaccgtattatttcttgtatgtaccaatggattagatttgtgtgcattaataataacgagctg
gttggctggctaggcagcagaaaatgaattaaaatggctggctttgtcgatcaagcatctttatccgt
tcattcccatgattggacccacttctgcctgcaacttcatcactgtccctacacaacattacctacca
ccaccagcaccaccggctctggcagttccccgtttccatttctttgaaagatctttgttttcatcctt
ttttagcttgatttttaagttatttgtgcacagttcatttcacacaagtaactagttggagattaagt
ccgtcagaagagtctgtttcgcattgagtttaaaggttaaaaaagtatttttcagaaaaaaatatcat
gtttaaaaaaaaaagcaatatgtgttcttgacaaaattaaatttaaatatttttaaataatattta
aaaagtgtttttaaaatatgagtgctgcttaactttcctctcgttgcttgtttgaaattatcattttc
tagtagaatatactagaaatcttttaaattaccataatacccttagcccgcaaatctctcttattaaa
ttcttaatctttattaaagatattataagacaatgaaaaaattattaaaatatttactgcgtgcccag
ctatttgtttctttttttttttttaatttttttttttattgcagggttgttgcaatgactttttcaat
tttccccttgtttcttacactttttctctataaattttcaccaattcttctctttcaatcaccccac
ttcattgcgtttcccttctttattctccttttttgcgtttggttagtctattttctcttttattttct
cttttattttctgtttgcttcttatttttcccaagatttttggtttttgagtgttctgctttagttgg
cttgcatacaagcctacaaggctctggagatctgctaaggactgtagttgtttcccttgatcatggcg
ttttactcttgctgcttatataataacttgctcaggctggcctagagaaaatttgcatatcatggcac
tattagtgttcaaaatttgattatcatgtaataagcggcttgatctgtggtttaaaagtttgtactca
acttacaacgtcatgatgctttttcttggtgactatgttgttctcataaggccaactttactagtttt
ctttctaatggccgagttccttttcaacttaaatggcggaatagttcagctctaaatttctttcttg
ttgtcaaaatttcttatctatttctgggtcaaaatatttaactttccacgaagaagtttcaagctt
ccttcttttgatggttcctgcatatgtgtttcttggagaattattctctctactctgctttattcttc
ttgcagggaagtctttcctttggtcaatagaaaaaaacagttctttcttctttgatcaacccatttt
taaatgggaattttttttttctgctgtatcccacagctcttttaaaaataaacccattattgatattg
gatctttattttgcacaaggttctgattctgttgggcactctttgctaaattgtttaattatgtgtgc
agtgatctatagatctttaacatttatgtgctgcattatttaagacagtgttcttagtcaacagtaca
ctcttaagtctctaaatcaaatataaaatcaaaatggtcggcgaggtggtaggtgtagattttttttt
tactcttctagaatcactggttttgttgggttttgactttagggtgattatccaaatgcttatatact
gatattttcagttcgttttaatatatccgtttttgtgattcttacatttgtttttgtttcatctgaac
tcttgcagaggttttctgatcataagct

Arabidopsis thaliana SUCROSE-PROTON SYMPORTER 2

gcatgcaaaatagcacaccatttatgtttatattttcaaattatttaatacatttcaatatttcataa
gtgtgatttttttttttttttgtcaatttcataagtgtgatttgtcatttgtattaaacaattgtatcg
cgcagtacaaataaacagtgggagaggtgaaaatgcagttataaaactgtccaataatttactaacac
atttaaatatctaaaaagagtgtttcaaaaaaaattcttttgaaataagaaaagtgatagatattttt
acgctttcgtctgaaaataaaacaataatagtttattagaaaaatgttatcaccgaaaattattctag
tgccactcgctcggatcgaaattcgaaagttatattctttctctttacctaatataaaaatcacaaga
aaaatcaatccgaatatatctatcaacatagtatatgcccttacatattgtttctgactttctctat
ccgaatttctcgcttcatggttttttttttaacatattctcatttaattttcattactattatataact

aaaagatggaaataaaataaagtgtctttgagaatcgaacgtccatatcagtaagatagtttgtgtga
aggtaaaatctaaaagatttaagttccaaaaacagaaaataatatattacgctaaaaaagaagaaaat
aattaaatacaaaacagaaaaaaataatatacgacagacacgtgtcacgaagataccctacgctatag
acacagctctgttttctcttttctatgcctcaaggctctcttaacttcactgtctcctcttcggataa
tcctatccttctcttcctataaatacctctccactcttcctcttcctccaccactacaaccaccgcaa
caaccaccaaaaaccctctcaaagaaatttcttttttttcttactttcttggtttgtcaaat
